# The Odilorhabdin Antibiotic Biosynthetic Cluster and Acetyltransferase Self-Resistance Locus Are Niche and Species Specific

**DOI:** 10.1128/mbio.02826-21

**Published:** 2022-01-11

**Authors:** Anne Lanois-Nouri, Lucile Pantel, Jun Fu, Jessica Houard, Jean-Claude Ogier, Yury S. Polikanov, Emilie Racine, Hailong Wang, Sophie Gaudriault, Alain Givaudan, Maxime Gualtieri

**Affiliations:** a DGIMI, Univ Montpellier, INRAE, Montpellier, France; b Nosopharm, Nîmes, France; c State Key Laboratory of Microbial Technology, Institute of Microbial Technology, Helmholtz International Lab for Anti-infectives, Shandong University –Helmholtz Institute of Biotechnology, Qingdao, Shandong, China; d Department of Biological Sciences, University of Illinois at Chicago, Chicago, Illinois, USA; e Department of Pharmaceutical Sciences, University of Illinois at Chicago, Chicago, Illinois, USA; Indiana University Bloomington

**Keywords:** BGC locus cloning, antibiotic resistance, genomics and phylogenetic analysis, invertebrate-microbe interactions, peptide modification

## Abstract

Antibiotic resistance is an increasing threat to human health. A direct link has been established between antimicrobial self-resistance determinants of antibiotic producers, environmental bacteria, and clinical pathogens. Natural odilorhabdins (ODLs) constitute a new family of 10-mer linear cationic peptide antibiotics inhibiting bacterial translation by binding to the 30S subunit of the ribosome. These bioactive secondary metabolites are produced by entomopathogenic bacterial symbiont *Xenorhabdus* (*Morganellaceae*), vectored by the soil-dwelling nematodes. ODL-producing Xenorhabdus nematophila symbionts have mechanisms of self-protection. In this study, we cloned the 44.5-kb *odl* biosynthetic gene cluster (*odl*-BGC) of the symbiont by recombineering and showed that the *N*-acetyltransferase-encoding gene, *oatA*, is responsible for ODL resistance. *In vitro* acetylation and liquid chromatography-tandem mass spectrometry (LC-MS/MS) analyses showed that OatA targeted the side chain amino group of ODL rare amino acids, leading to a loss of translation inhibition and antibacterial properties. Functional, genomic, and phylogenetic analyses of *oatA* revealed an exclusive *cis*-link to the odilorhabdin BGC, found only in *X. nematophila* and a specific phylogenetic clade of *Photorhabdus*. This work highlights the coevolution of antibiotic production and self-resistance as ancient features of this unique tripartite complex of host-vector-symbiont interactions without *odl*-BGC dissemination by lateral gene transfer.

## INTRODUCTION

Bioactive natural products and secondary metabolites are a major source of novel antibiotics. Most of the natural drugs identified in the last 40 years originate from soil and marine bacteria, such as actinomycetes in particular (1). Antibacterial products are ancient, and the origins of biosynthetic gene clusters (BGCs) encoding polyketide molecules, such as erythromycin, may date back to 800 million years ago (2). Logically, antimicrobial resistance may, therefore, be just as ancient.

Bacteria carrying BGCs encoding antimicrobial agents require protective mechanisms against these compounds (3), to ensure that their production leads to the destruction of other susceptible microorganisms without causing suicide of the producing bacterium. Over time, a copy of the self-resistance determinant integrates into the BGC, leading to coregulated expression, often through incorporation into the same operon. It has been suggested that horizontal gene transfer (HGT) of entire groups of genes plays a role in the evolution of BGCs and resistance. Over time, bacteria acquire resistance elements reflecting their current and past encounters with antibiotic producers (4). It has become clear that the environment is the source of an almost unlimited diversity of antibiotic resistance elements that are both extremely specific (e.g., inactivating enzymes) and broad (e.g., efflux systems), enabling the bacterium to counteract most antibiotics. Modification of the antibiotic target is one of the ways in which bacteria protect themselves from the lethal effects of their own antibiotics. Saccharopolyspora erythraea, which produces erythromycin, also produces an enzyme that attaches a methyl group to a specific site on the 23S rRNA, reducing the affinity of ribosomes for this antibiotic (5). Another mechanism of resistance involves modification of the antibiotic. Some aminoglycoside (AG)-producing actinomycete strains also produce enzymes that inactivate this class of antibiotics by adding phosphate, acetate, or adenylate groups (6). Brevibacillus brevis uses an *N*-acetyltransferase to modify the antimicrobial peptide edeine (EDE) (7).

**FIG 1:**
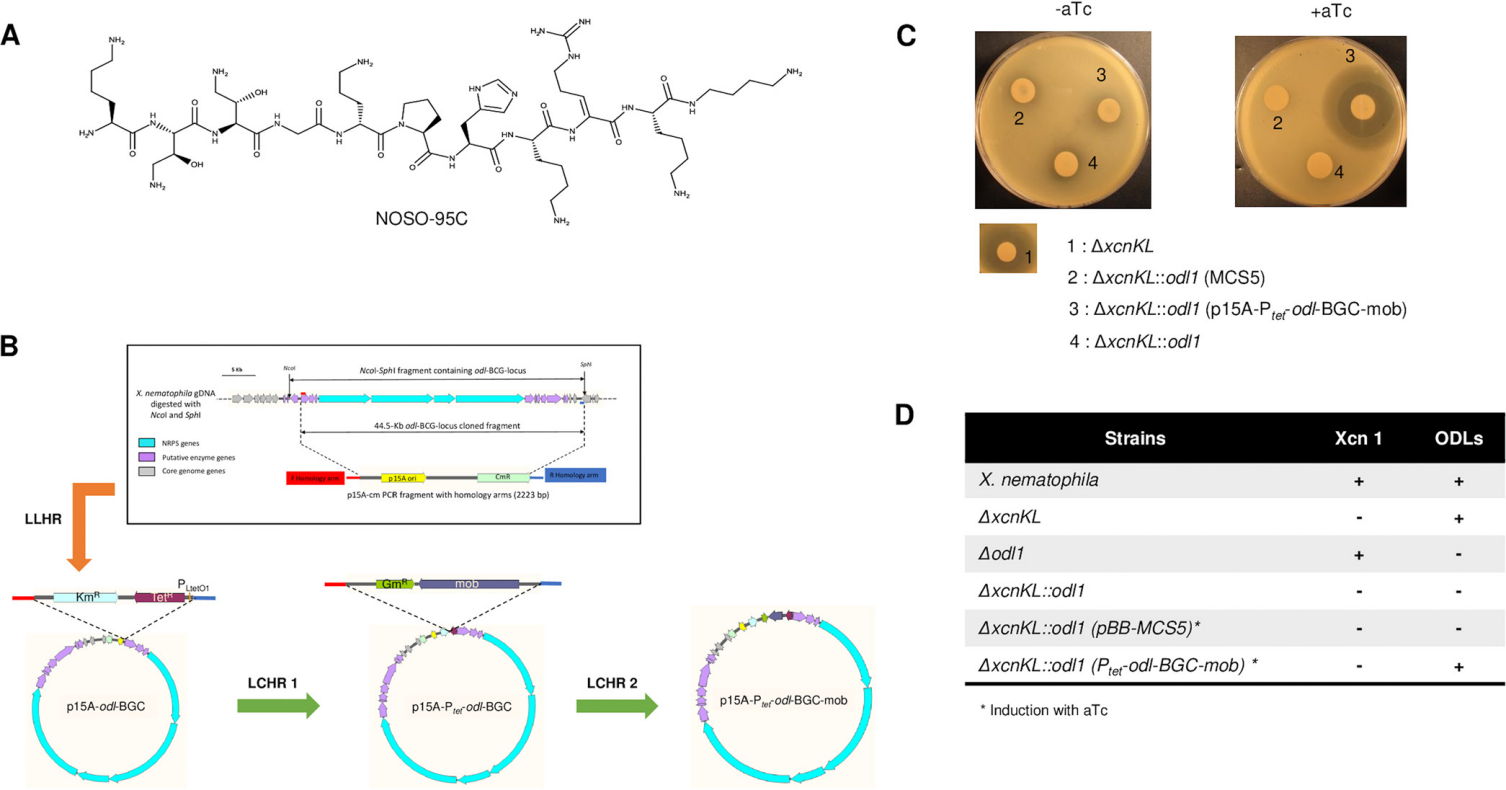
NOSO-95C structure (A), recombineering construction of the *X. nematophila odl*-BGC locus (B), and complementation in *Xenorhabdus* mutants (C and D). (A) Chemical structure of NOSO-95C. l-Lysine (Lys_1_, Lys_8_, and Lys_10_) (2*S*,3*S*)-α,γ-diamino β-hydroxybutyric acid [Dab(βOH)_2_], (2*S*,3*S*)-α,γ-2,4-diamino β-hydroxybutyric acid [Dab(βOH)_3_], glycine (Gly_4_), d-ornithine (Orn_5_), proline (Pro_6_), histidine (His_7_), Z-α,β-dehydroarginine (Dha_9_), and a functionalized secondary amide at the C-terminal position (α,δ-diamino butane [Dbt_11_]) are shown. (B) Recombineering construction of the *odl*-BGC locus. Three-step recombineering construction of p15A-P*_tet_*-*odl*-BGC-mob is shown. Linear-plus-linear homologous recombination (LLHR) between the 44.5-kb *odl*-BGC fragment and the p15A origin vector generated p15A-*odl*-BGC. A first linear-plus-circular homologous recombination (LCHR1) between the Km^r^-Tet^r^-P_LtetO1_ fragment and the p15A-*odl*-BGC plasmid generated p15A-P*_tet_*-*odl*-BGC containing the 44.5-kb *odl*-BGC fragment under the control of the P_LtetO1_ promoter. Finally, a second linear-plus-circular homologous recombination (LCHR2) between the Gm^r^-*mob* fragment and the p15A-P*_tet_*-*odl*-BGC plasmid generated the mobilizable plasmid p15A-P*_tet_*-*odl*-BGC-mob. Complementation was done in *Xenorhabdus* mutants. (C) Global antimicrobial activities against Klebsiella pneumoniae ATCC 43816 as a target of strains harboring the *odl*-BGC locus. After 24 h at 37°C, zones of growth inhibition were observed. (D) Production and LC-MS analysis of secondary metabolites (Xcn 1, xenocoumacin 1; ODLs, odilorhabdins) in complemented strains (Fig. S1) at 210 nm.

The entomopathogenic bacteria from the genera *Xenorhabdus* and *Photorhabdus* were formerly assigned to the *Enterobacteriaceae* but have recently been reclassified as members of the *Morganellaceae* (8). These two genera form tight sister groups, and their members have specific and persistent mutualistic relationships with the infective juveniles (IJs) of soil nematodes (Rhabditida: Steinernematidae and Heterorhabditidae, respectively). These entomopathogenic nematodes (EPNs) cooperate with *Xenorhabdus* and *Photorhabdus* symbionts to kill a broad range of soil-dwelling insect larval hosts (9). It has been suggested that the ancestors of the symbiotic bacteria of EPNs arose due to ecological events about 375 million years ago and that they provided EPNs with the opportunity to colonize new nematode hosts (10). *Photorhabdus* and *Xenorhabdus* are both obligate symbionts of EPNs. Indeed, EPN-symbiotic bacteria have never been found living freely in soil, although metagenomics studies have recently detected their presence in a minority microbiota associated with the production of insect larvae (11). However, *Photorhabdus* has been isolated in a dozen cases of soft tissue abscesses and disseminated bacteremia in humans in the United States and Australia (12, 13). It has become evident that as long suspected, *Xenorhabdus* and *Photorhabdus* are excellent sources of novel antimicrobial metabolites (14). EPN-symbiotic bacteria have a complex secondary metabolism characterized by the production of large amounts of specialized secondary metabolites, such as polyketides (PKs) and nonribosomal peptides (NRPs) (15). Many bioactive metabolites from diverse chemical classes with nematicidal, antifungal, or antibacterial activity have been reported (16). It has been suggested that the antimicrobial compounds produced by EPN-symbiotic bacteria kill foreign bacterial competitors, to protect the cadavers of the insects in which they multiply from invasion by other microbes (14). Two novel lead compounds from EPN-symbiotic bacteria are currently in pipelines for the development of novel classes of antibiotics for treating multidrug-resistant Gram-negative bacterial infections (17). The first of these classes is the odilorhabdins (ODLs), NRP secondary metabolites from *Xenorhabdus* (18). The second corresponds to darobactin A, a natural product discovered by screening *Photorhabdus* symbionts directly targeting an integral outer membrane protein (19). ODLs, the first-in-class antibiotics, were discovered, as part of a program aiming to renew the therapeutic antibacterial arsenal, by screening a collection of *Xenorhabdus* species for antimicrobial activity (18, 20). NOSO-95A, -B, and -C, natural ODLs, were isolated from Xenorhabdus nematophila K102 (18). They are 10-mer linear cationic peptides containing six proteinogenic and four to six nonproteinogenic amino acids (Fig. 1A), with promising broad-spectrum activity against both Gram-positive and Gram-negative bacteria, including carbapenem-resistant *Enterobacteriaceae* (CRE) and methicillin-resistant Staphylococcus aureus (MRSA) (21). Efforts to develop this new antimicrobial class have been pursued: NOSO-502 is the first candidate from the ODL family to undergo preclinical development for the treatment of multidrug-resistant *Enterobacteriaceae* infections (22, 23). The members of the ODL family have bactericidal action through the specific inhibition of bacterial translation resulting from their interaction with the 30S ribosomal subunit at a site not exploited by other clinically useful antibiotics. They bind to the ribosome in the vicinity of the decoding center (DC), interacting simultaneously with the nucleotides of the 16S rRNA and the anticodon loop of the incoming A-site aminoacyl-tRNA. At low concentrations, ODLs cause the misincorporation of amino acids by decreasing decoding fidelity, whereas at high concentrations, they interfere with the progression of the ribosome along the mRNA. The ability of ODLs to cause miscoding is probably responsible for their bactericidal activity (18, 24). As expected, given that ODLs contain nonproteinogenic amino acids synthesized by nonribosomal peptide synthetases (NRPSs), the putative BGC contains four large NRPS-encoding genes (*odl1*, *odl2*, *odl3*, and *odl4*) (18). Only a few mutant bacteria resistant to ODLs have been reported to date. For example, the His56Tyr variant of the ribosomal protein S10 confers a high degree of resistance to ODLs and has been identified in Escherichia coli (18). The S10 gene is located close to the 16S rRNA gene. A similar resistance profile was observed for Klebsiella pneumoniae strains bearing mutations affecting the CrrAB two-component system (TCS) (22, 25). CrrB mutations cause NOSO-502 resistance by upregulating the KexD efflux pump. This system is also known to decrease colistin sensitivity (25).

10.1128/mBio.02826-21.1 FIG S1 LC-MS analysis of *X. nematophila* wild type (WT), mutants, and complemented strains for the production of odilorhabdins (NOSO-95A, -B, and -C) and xenocoumacin. Supernatants from 96-h cultures (or 78-h cultures from complemented strains) were filtered through 0.2-μm filters and analyzed as previously described in Materials and Methods in the main text by LC-MS at 210 nm with molecular masses from 630 to 650 (for ODLs) and from 465 to 467 (for Xcn 1) after extracted ion chromatographic (EIC) analysis. Download FIG S1, PDF file, 1.2 MB. Copyright © 2022 Lanois-Nouri et al. 2022 Lanois-Nouri et al. https://creativecommons.org/licenses/by/4.0/ This content is distributed under the terms of the Creative Commons Attribution 4.0 International license.

We show here that ODL-producing *X. nematophila* strains display high levels of resistance to their own ODL antibiotic. We investigated the mechanism of self-resistance by cloning the *odl*-BGC. We also assessed the possible involvement of genes flanking this cluster in resistance. A Gcn5-related *N*-acetyltransferase (GNAT)-encoding gene, *oatA*, was identified as responsible for resistance. *In vitro* acetylation and liquid chromatography-tandem mass spectrometry (LC-MS/MS) analyses were performed to identify the target of the enzyme. Multiacetylated forms of NOSO-95C lost their antibacterial activity and were unable to inhibit translation in an *in vitro* translation assay, revealing the molecular mechanism of self-resistance. A molecular study of the distribution of *oatA* revealed that this self-resistance gene was *cis*-linked to the odilorhabdin BGC and found only in *X. nematophila* and a single phylogenetic clade of *Photorhabdus* formerly classified as the species Photorhabdus luminescens.

## RESULTS AND DISCUSSION

### Self-resistance of the odilorhabdin-producing strain Xenorhabdus nematophila K102.

The supernatants of 80 cultured strains of various *Xenorhabdus* species were screened for the presence of antibacterial activity (18). Three antibacterial metabolites were characterized from *X. nematophila* strain K102 (CNCM I-4530): NOSO-95A (MW: 1,296 Da), NOSO-95B (MW: 1,280 Da), and NOSO-95C (MW: 1,264 Da) (18). These compounds have similar structures, differing only by the presence of one or two hydroxylysines in place of the lysine residues in positions 8 and 10 (Fig. 1A). NOSO-95C was the first of these compounds for which total chemical synthesis was completed, and this compound was, therefore, chosen for this study. It was active against a range of clinically relevant Gram-negative bacteria, with a MIC of ≤8 μg/mL (Table 1). Interestingly, NOSO-95C was less active against some members of the *Morganellaceae* family, such as Providencia stuartii ATCC 29914 and Proteus mirabilis ATCC 29906, with MICs of 16 μg/mL and 32 μg/mL, respectively, and was inactive against other members of this family, such as Morganella morganii DSM 30164 and the ODL-producing strain *X. nematophila* K102, with a MIC of >2,048 μg/mL, confirming that ODL producers develop mechanisms of self-resistance to ODLs (Table 1).

**TABLE 1:** MICs of NOSO-95C against a panel of Gram-negative species^*a*^

Strain	Family	MIC of NOSO-95C (μg/mL)
Escherichia coli ATCC 25922	*Enterobacteriaceae*	8
Klebsiella pneumoniae NC 09633	*Enterobacteriaceae*	8
Klebsiella aerogenes DSM 30053	*Enterobacteriaceae*	4
Enterobacter cloacae DSM 14563	*Enterobacteriaceae*	4
Citrobacter freundii DSM 30039	*Enterobacteriaceae*	4
Serratia marcescens DSM 17174	*Enterobacteriaceae*	8
Pseudomonas aeruginosa DSM 1117	*Pseudomonadaceae*	8
Acinetobacter baumannii ATCC 19606	*Moraxellaceae*	8
Proteus mirabilis ATCC 29906	*Morganellaceae*	32
Providencia stuartii ATCC 29914	*Morganellaceae*	16
Morganella morganii DSM 30164	*Morganellaceae*	>2,048
Xenorhabdus nematophila K102 (CNCM I-4530)	*Morganellaceae*	>2,048

a MICs were determined according to CLSI guidelines.

### Identification of a 44.5-kb locus in *X. nematophila* carrying a BGC encoding odilorhabdins.

Bioinformatic prediction and the inactivation of an NRPS gene (24) led to a 32.5-kb BGC locus encompassing *odl1* to *odl4* being considered to encode native odilorhabdin products. This cluster consists of four giant NRPS-encoding genes in the genomic sequence of *X. nematophila* (Fig. 1B). One challenging way to demonstrate that the complete set of genes embedded within a cluster is required for the biosynthesis of a particular metabolite is to insert the BGC locus into a heterologous host. Considerable amounts of genomic data are available for EPN-symbiotic bacteria, which have unexplored pathways of secondary metabolism (26, 27). Optimized protocols have, therefore, been developed for the efficient cloning of large DNA fragments (27, 28). We obtained a mobilizable plasmid carrying genes encoding NRPS putatively required for odilorhabdin synthesis, through a three-step cloning procedure with one linear-linear homologous recombination (LLHR) step and two linear-circular homologous recombination (LCHR) steps involving RecET- and Redαβ-based techniques, respectively (29) (Fig. 1B). The cloned 44.5-kb *odl-*BGC locus from *X. nematophila* under P*_tet_* control extended from the start of the *ectB* gene to the end of the following SphI site (Fig. 1B). In *X. nematophila*, xenocoumacin (Xcn) production (Δ*xcnKL* mutant strain) has been shown to be responsible for the principal zone of growth inhibition in the target strain Micrococcus luteus, and the double mutant strain Δ*xcnKL*::*odl1* (previously called Δ*xcnKL*::F) has been shown to display an almost complete loss of this zone of inhibition (18, 30). We show here that the complementation of the *X. nematophila* double mutant strain (Δ*xcnKL*::*odl1*) to full functionality through the introduction of a plasmid harboring the *X. nematophila* 44.5-kb *odl-*BGC locus under the control of P*_tet_* restored antibacterial activity against Klebsiella pneumoniae (Fig. 1C). In addition, our liquid chromatography coupled to mass spectrometry (LC-MS) analysis revealed the presence of 1,296-Da (NOSO-95A), 1,280-Da (NOSO-95B), and 1,264-Da (NOSO-95C) products in the exconjugants (Fig. 1D; see also Fig. S1 in the supplemental material).

We then introduced the p15A-P*_tet_-odl-*BGC plasmid DNA into an E. coli strain carrying the phosphopantheteinyl transferase (PPTase) gene (*ngrA*) of *X. nematophila* (30). PPTases posttranslationally modify the modular and iterative synthases acting in a processive fashion: fatty acid synthases (FAS), polyketide synthases (PKS), and NRPS (31). We assessed antibiotic production under predefined conditions in which *odl* mutant complementation had been obtained. We were able to detect the expression of *ectB* (the first gene of the cloned locus) and *odl1* (the first NRPS gene) by quantitative reverse transcription PCR (RT-qPCR) experiments (Fig. S2A), but no antibiotic activity against K. pneumoniae (Fig. S2B) or odilorhabdin derivatives were detected in the extracts of E. coli transformants by LC-MS profiling (Fig. S1 and S2C). The cluster encompassing *odl1* to *odl4* from Photorhabdus laumondii TT01 had been cloned in a previous study, but again, no derivative products were detected (29). The extent to which a pathway is successfully expressed is unpredictable, due to the idiosyncrasies of the different heterologous bacterial hosts, which may not necessarily produce all the necessary precursors (31). For example, the antibiotic erythromycin C is produced in 10,000 times larger amounts by its native host *S. erythraea* than by the heterologous host E. coli K207-2 (32). These data suggest that the 44.5-kb *odl* locus may lack modification enzyme genes in E. coli.

10.1128/mBio.02826-21.2 FIG S2 Complementation of E. coli XL1 (pBB-*ngrA*) with p15A-P*_tet_*-*odl*-BGC. (A) Expression of *odl*-BGC locus genes in XL1 (pBB-*ngrA*) (p15A-P*_tet_*-*odl*-BGC). Total RNA was first extracted from 100-mL broth cultures of XL1 (pBB-*ngrA*) (p15A-P*_tet_*-*odl*-BGC) or XL1 (pBB-*ngrA*) (pACYC184) incubated with or without aTc at 28°C for 78 h, as previously described (1). Briefly, RNA was isolated with the RNeasy Protect Bacteria miniprep kit (Qiagen), including DNase I incubation in accordance with the manufacturer’s recommendations. For each RNA preparation, we assessed DNA contamination by carrying out a 16S control PCR. The quantity and quality of total RNA were assessed with a NanoDrop 2000 spectrophotometer (Thermo Fisher Scientific) and an Agilent 2100 Bioanalyzer with the RNA 6000 Nano LabChip kit (Agilent). RT-qPCR was performed in two steps, as previously described (1). First, the cDNA was synthesized from 0.5 μg of total RNA with SuperScript II reverse transcriptase from Invitrogen and random hexamers (100 mg/L) from Promega. We then performed qPCR in triplicate with the SensiFAST SYBR *No-ROX* kit (Bioline), with 1 μL of cDNA synthesis mixture (diluted 1:50) and 1 μM specific primers for the studied genes (Table S2). The enzyme was activated by heating for 2 min at 95°C. All qPCRs were performed in three technical replicates, with 45 cycles of 95°C for 5 s and 61°C for 30 s, and were monitored in the LightCycler 480 system (Roche). Melting curves were analyzed for each reaction, and each curve contained a single peak. For standard curves, the amounts of PCR products generated were determined with serially diluted genomic DNA from *X. nematophila* (for *ectB* and *odl1*) or E. coli (for *gyrB*). Histograms represent the relative transcript levels of *ectB* and *odl1* versus the *gyrB* gene used as housekeeping gene, as calculated with LightCycler 480 software (Roche). The data shown are the medians of experimental triplicates, and error bars represent the statistical standard deviations. (B) Global antimicrobial activities against Klebsiella pneumoniae ATCC 43816 as a target of strains harboring *odl*-BGC loci (see Materials and Methods in the main text). (C) Supernatants from cultures used for RNA preparation (see panel A) were passed through a filter with 0.2-μm pores and concentrated 10-fold for LC-MS analysis. Natural odilorhabdins (NOSO-95A, -B, and -C) and xenocoumacin 1 (Xcn 1) were identified in culture supernatants by LC-MS as described in Materials and Methods in the main text (see also Fig. S1). Download FIG S2, PDF file, 0.1 MB. Copyright © 2022 Lanois-Nouri et al. 2022 Lanois-Nouri et al. https://creativecommons.org/licenses/by/4.0/ This content is distributed under the terms of the Creative Commons Attribution 4.0 International license.

### The *N*-acetyltransferase gene, *oatA*, embedded in the *odl*-BGC is responsible for NOSO-95C self-resistance.

**FIG 2:**
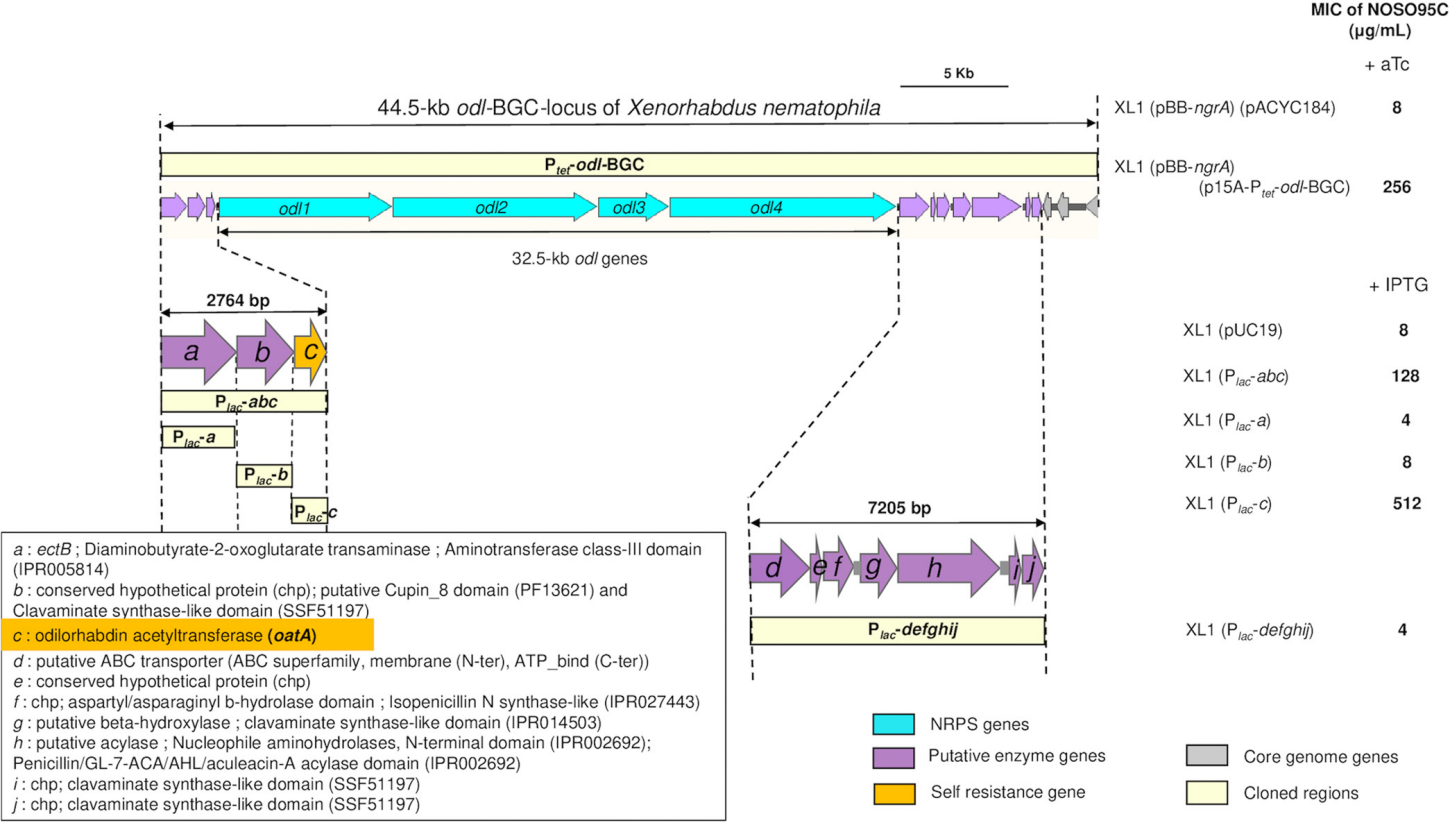
Subcloning of odilorhabdin BGC locus and putative ODL resistance genes. Odilorhabdin self-resistance gene identification was done by subcloning of the *odl*-BGC locus of *X. nematophila*. The *odl*-BGC locus of *X. nematophila* cloned into XL1 Blue MRF′ confers resistance to odilorhabdin. In order to identify the odilorhabdin self-resistance gene, subcloning of the three upstream genes and the seven downstream genes from the *odl*-BGC locus was realized by PCR amplification using appropriate primers into pUC19 vector under the control of the P*_lac_* promoter. On the right are MICs of NOSO-95C for XL1 Blue MRF′ carrying the entire cloned fragment after induction with aTc (200 ng/mL) or subcloned genes after induction with IPTG (0.2 mM). pACYC184 and pUC19 were used as controls.

As expected, the MICs obtained showed that the complete 44.5-kb *odl-*BGC locus of *X. nematophila* expressed under the control of P*_tet_* after anhydrotetracycline (aTc) induction encoded NOSO-95C resistance (MIC, 256 μg/mL) (Fig. 2). For identification of the genes responsible for odilorhabdin self-resistance, we also subcloned the genes at the periphery of the *odl*-BGC locus, giving 10 genes in all, in E. coli (Fig. 2). Only expression of the 2,764-bp DNA fragment containing the three genes upstream from *odl1* resulted in NOSO-95C resistance in the recombinant E. coli strains, and subcloning experiments clearly showed that a single gene, “*c*,” was responsible for resistance to NOSO-95C (MIC, 512 μg/mL) after isopropyl-β-d-thiogalactoside (IPTG) induction (Fig. 2). The BLASTP genome annotation and InterPro domain identified the C protein as a Gcn5-related *N*-acetyltransferase (GNAT). We therefore named this gene *oatA*, for odilorhabdin acetyltransferase A. The GNAT family includes more than 100,000 enzymes present in eukaryotes and prokaryotes (33). These enzymes catalyze the transfer of an acetyl group from acetyl coenzyme A (acetyl-CoA) to a primary amine in diverse acceptor molecules. They are involved in multiple physiological events, including bacterial drug resistance, the regulation of transcription, toxin-antitoxin system control, detoxification, and protection against oxidative stress (34). Many aminoglycoside (AG) acetyltransferases have since been described and are among the principal sources of clinically relevant aminoglycoside resistance (35).

### Characterization of *Xenorhabdus* OatA_Xn_ and its *N*-acetylated products.

OatA from *X. nematophila* F1 was first overexpressed in the BL21 system and purified (Fig. S3). We obtained about 2.2 mg of OatA::tag His per liter of culture. Assuming that ODLs are the substrates of this enzyme, *in vitro* acetylation experiments were performed to identify the sites acetylated. NOSO-95C was reacted with the purified OatA enzyme in the presence of acetyl-CoA. After incubation for 15 min, LC-MS revealed the presence of a single UV peak at a retention time of 9.71 min with a major associated *m/z* of 1,307, consistent with the mass of monoacetylated NOSO-95C (Fig. S4A). After incubation for 4 h or 24 h, we observed two separate UV peaks at retention times of 9.6 min and 10.4 min with associated *m/z* of 1,349 and 1,391, respectively, consistent with the masses of biacetylated and triacetylated NOSO-95C. The native compound (*m/z* 1,265) had entirely disappeared. The peak area ratios at 230 nm (biacetylated/triacetylated) were 25 and 4.2, respectively, indicating that the biacetylated form remained largely predominant. An autoacetylation control was performed, with NOSO-95C and acetyl-CoA in the absence of enzyme (Fig. S4B). After 15 min, 4 h, and 24 h, native unacetylated NOSO-95C was mainly detected, with 100%, 97%, and 72%, respectively, of the compound remaining (Fig. S4).

10.1128/mBio.02826-21.3 FIG S3 SDS-PAGE of His-tagged OatA protein production and purification. We assessed production of the His-tagged OatA protein, by centrifuging 500 mL of a culture of the strain BL21(DE3) (pET-*oatA*) after induction with IPTG and resuspending the pellet in 20 mL of 2× Laemmli sample buffer. The sample was then run on a 15% acrylamide SDS-PAGE gel (2). The BL21(DE3) (pET-28a) strain was used as a control (1). After purification of the His-tagged OatA protein with Ni-NTA agarose, aliquots (10 mL) of 250 mM imidazole (3) and 500 mM imidazole (4) eluates were also run on a 15% acrylamide SDS-PAGE gel with the PageRuler Plus prestained protein ladder (Thermo Fisher Scientific) (M). After electrophoresis at 150 V (3 W) for 5 h, the acrylamide gel was stained overnight in a solution containing 0.1% Coomassie Blue with ethanol-acetic acid (40% to 10%) and destained in ethanol-acetic acid (40% to 10%) for 3 h before the gel was scanned. Download FIG S3, PDF file, 0.3 MB. Copyright © 2022 Lanois-Nouri et al. 2022 Lanois-Nouri et al. https://creativecommons.org/licenses/by/4.0/ This content is distributed under the terms of the Creative Commons Attribution 4.0 International license.

10.1128/mBio.02826-21.4 FIG S4 LC-MS analysis of NOSO-95C and acetylated derivatives after *in vitro* acetylation (see Materials and Methods in the main text) at different times of reaction (15 min, 4 h, or 24 h) with acetyl-CoA (B), odilorhabdin acetyltransferase A (OatA) (C), or both (A). For each tested condition, a table and corresponding LC-MS chromatograms present observed ions with retention time, peak area, and suggested compounds. LC-MS was performed on an Agilent 1260 Infinity HPLC system with a Waters Symmetry analytical C_18_ column (5 μm, 4.6 mm by 150 mm). We injected 10 μL of reaction mixture into the apparatus, the flow rate was set to 0.7 mL/min, and UV detection was performed at 220, 230, and 250 nm. The following mobile phases were used: A, 0.1% trifluoroacetic acid in water; B, acetonitrile. The following gradient was used: 2 to 30% of mobile phase B in mobile phase A from 0 to 15 min. ESI-LC-MS data were obtained in positive mode on an Agilent 1260 Infinity system (Agilent 6120 quadrupole LC/MS, 1260 quaternary pump, 1260 ALS, 1260 TCC, 1260 DAD VL, 1260 FC-AS). Download FIG S4, PDF file, 0.6 MB. Copyright © 2022 Lanois-Nouri et al. 2022 Lanois-Nouri et al. https://creativecommons.org/licenses/by/4.0/ This content is distributed under the terms of the Creative Commons Attribution 4.0 International license.

**FIG 3:**
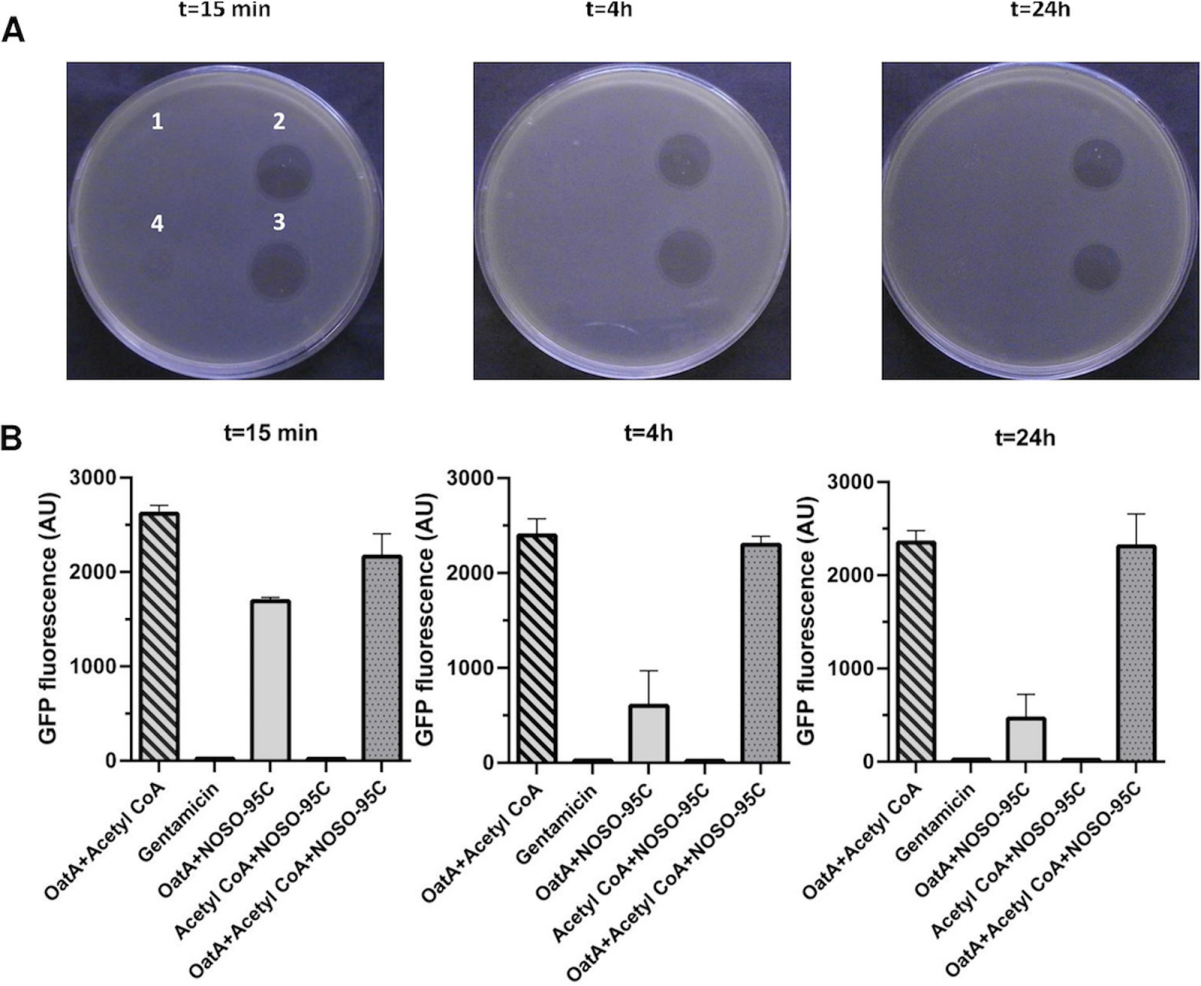
Characterization of acetylated NOSO-95C. (A) Antibacterial activity against E. coli ATCC 25922 of NOSO-95C after various reaction times (15 min, 4 h, or 24 h) with acetyl-CoA (2), odilorhabdin acetyltransferase (OatA) (3), or both (4). OatA with acetyl-CoA was used as a negative control (1). (B) Inhibition of synthesis of the GFP reporter protein in the E. coli cell-free transcription-translation system by NOSO-95C after various reaction times (15 min, 4 h, or 24 h) with acetyl-CoA, OatA, or both. OatA with acetyl-CoA was used as a negative control. Gentamicin (10 μM) was used as a positive control. The data shown are the means of three experiments ± SD.

Whatever the incubation time, reaction mixtures lost antibacterial activity, whereas the controls did not, indicating that the mono-, bi-, and triacetylated forms of NOSO-95C were inactive (Fig. 3A). Previous studies have shown that NOSO-95C interferes with protein synthesis in living bacteria and inhibits the production of green fluorescent protein (GFP) in an E. coli cell-free transcription-translation assay (18). We performed *in vitro* translation assays to demonstrate that the loss of antibacterial activity in acetylated compounds was due to a loss of their ability to inhibit bacterial translation. Gentamicin, a ribosomal inhibitor, was used as a positive control; it blocked GFP translation, resulting in low levels of fluorescence. Similar inhibition profiles were observed with the unmodified NOSO-95C controls. OatA enzyme alone, without acetyl-CoA, appeared to limit partially the potential of NOSO-95C to inhibit translation. We hypothesized that the enzyme had a strong affinity for NOSO-95C, decreasing the amount of free compound available. The reaction mixtures containing mono-, bi-, and triacetylated NOSO-95C had no effect on GFP translation, resulting in high levels of fluorescence. The acetylated forms of NOSO-95C prevented the inhibition of protein synthesis, probably by blocking ribosome binding (Fig. 3B).

### Structural analysis of monoacetylated, biacetylated, and triacetylated NOSO-95C.

OatA belongs to the GNAT superfamily, a group of enzymes catalyzing the transfer of an acetyl group from acetyl-CoA to a primary amine in diverse acceptor molecules. NOSO-95C contains eight primary amino groups (ε- and α-primary amines of lysine in position 1), Dab(βOH) in positions 2 and 3, d-Orn in position 5, l-Lys in positions 8 and 10, and Dbt at the C-terminal position, all of which are possible sites of *N*-acetylation by OatA. The location of the acetyl groups was assessed by LC-MS/MS fragmentation analyses comparing monoacetylated, biacetylated, and triacetylated NOSO-95C with native NOSO-95C (Fig. S5). The presence of an additional acetyl group adds 42 Da to the initial molecular weight. The fragmentation spectrum of NOSO-95C (*m/z* 1,265) was first generated, to identify characteristic fragment ions (Table 2). For monoacetylated NOSO-95C (*m/z* 1,307), ion fragments with *m/z* of 1,178 and 1,020.6 were obtained by the elimination of 129 Da and 287 Da, respectively, consistent with the loss of Lys_1_ and Lys_1_-acetylated Dab(βOH)_2_ (Table 2). The fragmentation spectrum of biacetylated NOSO-95C (*m/z* 1,349) revealed ion fragments with *m/z* of 1,220.7, 1,062.6, and 904.6 obtained by the elimination of 129 Da, 287 Da, and 445 Da, respectively, consistent with the loss of Lys_1_, Lys_1_-acetylated Dab(βOH)_2_, and Lys_1_-acetylated Dab(βOH)_2_-acetylated Dab(βOH)_3_ (Table 2). The fragmentation profiles of mono- and biacetylated NOSO-95C therefore revealed structures with acetyl groups located on the amino groups of the Dab(βOH) residues. For the triacetylated NOSO-95C (*m/z* 1,391), a fragmentation pattern similar to that for biacetylated NOSO-95C was obtained, with the loss of 129 Da, 287 Da, and 445 Da, indicating that Dab(βOH)_2_ and Dab(βOH)_3_ were acetylated. A difference of 258 Da between *m/z* 1,391 and *m/z* 1,132.6 was assigned to the loss of Lys_10_ and Dbt_11_, with an acetyl group. Fragment ions with *m/z* of 1,174.6 and 687.4 were useful for determining which of these two amino acids were acetylated. These fragment ions are generated by the elimination of a nonmodified Dbt_11_ (88 Da), making it possible to conclude that the last acetyl group is located on the primary amino group of Lys_10_ (Table 2).

**TABLE 2:** MS/MS fragmentation analysis of acetylated odilorhabdin NOSO-95C^*a*^

NOSO-95C form and ion	*m/z*	Neutral loss from previous ion	Attribution	Assigned structure
NOSO-95C (*m/z* 1,265)				
[M + 2H]2+	633			Lys-Dab(βOH)-Dab(βOH)-Gly-Orn-Pro-His-Lys-Dha-Lys-Dbt
y_10_	1,136.7	129	Lys_1_	Dab(βOH)-Dab(βOH)-Gly-Orn-Pro-His-Lys-Dha-Lys-Dbt
y_9_	1,020.6	116	Dab(βOH)_2_	Dab(βOH)-Gly-Orn-Pro-His-Lys-Dha-Lys-Dbt
	804.4	216 (128 + 88)	Lys_10_-Dbt_11_	Dab(βOH)-Gly-Orn-Pro-His-Lys-Dha
y_8_	904.6	116	Dab(βOH)_3_	Gly-Orn-Pro-His-Lys-Dha-Lys-Dbt
	688.4	216 (128 + 88)	Lys_10_-Dbt_11_	Gly-Orn-Pro-His-Lys-Dha
y_6_	733.5	171	Gly_4_-Orn_5_	Pro-His-Lys-Dha-Lys-Dbt
	517.3	216 (128 + 88)	Lys_10_-Dbt_11_	Pro-His-Lys-Dha
b_9_	1,048.6	216 (128 + 88)	Lys_10_-Dbt_11_	Lys- Dab(βOH)-Dab(βOH)-Gly-Orn-Pro-His-Lys-Dha
Monoacetylated NOSO-95C (*m/z* 1,307)				
[M + 2H]2+	654			Lys-Dab(βOH)-Dab(βOH)-Gly-Orn-Pro-His-Lys-Dha-Lys-Dbt
y_10_	1,178	129	Lys_1_	AcDab(βOH)-Dab(βOH)-Gly-Orn-Pro-His-Lys-Dha-Lys-Dbt
y_9_	1,020.6	158	AcDab(βOH)_2_	Dab(βOH)-Gly-Orn-Pro-His-Lys-Dha-Lys-Dbt
y_8_	904.6	116	Dab(βOH)_3_	Gly-Orn-Pro-His-Lys-Dha-Lys-Dbt
	688.4	216 (128 + 88)	Lys_10_-Dbt_11_	Gly-Orn-Pro-His-Lys-Dha
y_6_	733.5	171	Gly_4_-Orn_5_	Pro-His-Lys-Dha-Lys-Dbt
	517.3	216 (128 + 88)	Lys_10_-Dbt_11_	Pro-His-Lys-Dha
y_5_	635.9	97	Pro_9_	His-Lys-Dha-Lys-Dbt
b_9_	1,090.6	216 (128 + 88)	Lys_10_-Dbt_11_	Lys-AcDab(βOH)-Dab(βOH)-Gly-Orn-Pro-His-Lys-Dha
Biacetylated NOSO-95C (*m/z* 1,349)				
[M + 2H]2+	675			
y_10_	1,220.7	129	Lys_1_	AcDab(βOH)-AcDab(βOH)-Gly-Orn-Pro-His-Lys-Dha-Lys-Dbt
y_9_	1,062.6	158	AcDab(βOH)_2_	AcDab(βOH)-Gly-Orn-Pro-His-Lys-Dha-Lys-Dbt
	846.4	216 (128 + 88)	Lys_10_-Dbt_11_	AcDab(βOH)-Gly-Orn-Pro-His-Lys-Dha
y_8_	904.6	158	AcDab(βOH)_3_	Gly-Orn-Pro-His-Lys-Dha-Lys-Dbt
	688.4	216 (128 + 88)	Lys_10_-Dbt_11_	Gly-Orn-Pro-His-Lys-Dha
y_6_	733.5	171	Gly_4_-Orn_5_	Pro-His-Lys-Dha-Lys-Dbt
	517.3	216 (128 + 88)	Lys_10_-Dbt_11_	Pro-His-Lys-Dha
b_9_	1,132.6	216 (128 + 88)	Lys_10_-Dbt_11_	Lys-AcDab(βOH)-AcDab(βOH)-Gly-Orn-Pro-His-Lys-Dha
Triacetylated NOSO-95C (*m/z* 1,391)				
[M + 2H]2+	696			
y_10_	1,262.7	129	Lys_1_	AcDab(βOH)-AcDab(βOH)-Gly-Orn-Pro-His-Lys-Dha-AcLys-Dbt
	1,174.6	88	Dbt	AcDab(βOH)-AcDab(βOH)-Gly-Orn-Pro-His-Lys-Dha-AcLys
y_9_	1,104.6	158	AcDab(βOH)_2_	AcDab(βOH)-Gly-Orn-Pro-His-Lys-Dha-AcLys-Dbt
y_8_	946.6	158	AcDab(βOH)_3_	Gly-Orn-Pro-His-Lys-Dha-AcLys-Dbt
y_6_	775.5	171	Gly_4_-Orn_5_	Pro-His-Lys-Dha-AcLys-Dbt
	687.4	88	Dbt	Pro-His-Lys-Dha-AcLys
	517.3	170	AcLys_10_	Pro-His-Lys-Dha
b_9_	1,132.6	258 (170 + 88)	AcLys_10_-Dbt_11_	Lys-AcDab(βOH)-AcDab(βOH)-Gly-Orn-Pro-His-Lys-Dha

a See also Fig. S5. For bi- and triacetylated NOSO-95C, structures were retroactively assigned. Abbreviation: Ac, acetyl group.

10.1128/mBio.02826-21.5 FIG S5 Fragmentation (MS/MS) spectrum of NOSO-95C (*m/z* 1,265) (A), monoacetylated NOSO-95C (*m/z* 1,307) (B), biacetylated NOSO-95C (*m/z* 1,349) (C), and triacetylated NOSO-95C (*m/z* 1,391) (D). After *in vitro* acetylation, LC-MS/MS was performed as described in Materials and Methods in the main text. Download FIG S5, PDF file, 0.3 MB. Copyright © 2022 Lanois-Nouri et al. 2022 Lanois-Nouri et al. https://creativecommons.org/licenses/by/4.0/ This content is distributed under the terms of the Creative Commons Attribution 4.0 International license.

The amino group of Dab(βOH)_2_ of NOSO-95C was the principal group acetylated by OatA, followed by Dab(βOH)_3_ and, to a lesser extent, Lys_10_. Within 15 min, NOSO-95C was totally metabolized by OatA, generating a 100% monoacetylated form modified exclusively at Dab(βOH)_2_ (Fig. S4A). Given the complete loss of antibacterial activity in this modified compound, we hypothesized that the amino group of Dab(βOH)_2_ was the main target of OatA. We tested this hypothesis by synthesizing NOSO-95179, in which Dab(βOH)_3_ was replaced by an Ala residue and Lys_10_-Dbt_11_ was removed. This compound had a MIC against E. coli XL1 (P*_lac_*-*oatA-Xn*) more than 512 times higher than that of the parental strain XL1 (pUC19), demonstrating that the presence of Dab(βOH)_3_ and Lys_10_ is not essential for the mechanism of resistance via acetylation. NOSO-95179 was then reacted with purified OatA in the presence of acetyl-CoA. After 24 h of incubation, LC-MS/MS analysis confirmed the presence of the Dab(βOH)_2_ monoacetylated form only. After 24 h of incubation with acetyl-CoA and OatA, NOSO-95179 also displayed a loss of antibacterial activity and of protein synthesis inhibition capacity (Fig. S6).

10.1128/mBio.02826-21.6 FIG S6 Characterization of OatA activity against a NOSO-95179 analog. (A) LCMS analysis of NOSO-95179 (*m/z* 1,022) acetylated derivatives after 24 h of reaction with acetyl-CoA and odilorhabdin acetyltransferase A (OatA): the form present was 100% monoacetylated (*m/z* 1,064). (B) MS/MS fragmentation analysis of monoacetylated NOSO-95179 (*m/z* 1,064): acetylation of the amine group on Dab(βOH)_2_. (C) Antibacterial activity assay for NOSO-95179 after 24 h of reaction with acetyl-CoA (1), and OatA (2), or both (3) against E. coli ATCC 2592. (D) Inhibition of synthesis of the GFP reporter protein in the E. coli cell-free transcription-translation system by NOSO-95179 after 24 h of reaction with acetyl-CoA, OatA, or both. Gentamicin (10 μM) was used as a positive control. The data shown are the means of three experiments ± SD. Download FIG S6, PDF file, 0.1 MB. Copyright © 2022 Lanois-Nouri et al. 2022 Lanois-Nouri et al. https://creativecommons.org/licenses/by/4.0/ This content is distributed under the terms of the Creative Commons Attribution 4.0 International license.

### Structural basis for the poor antimicrobial activity of acetylated ODLs.

**FIG 4:**
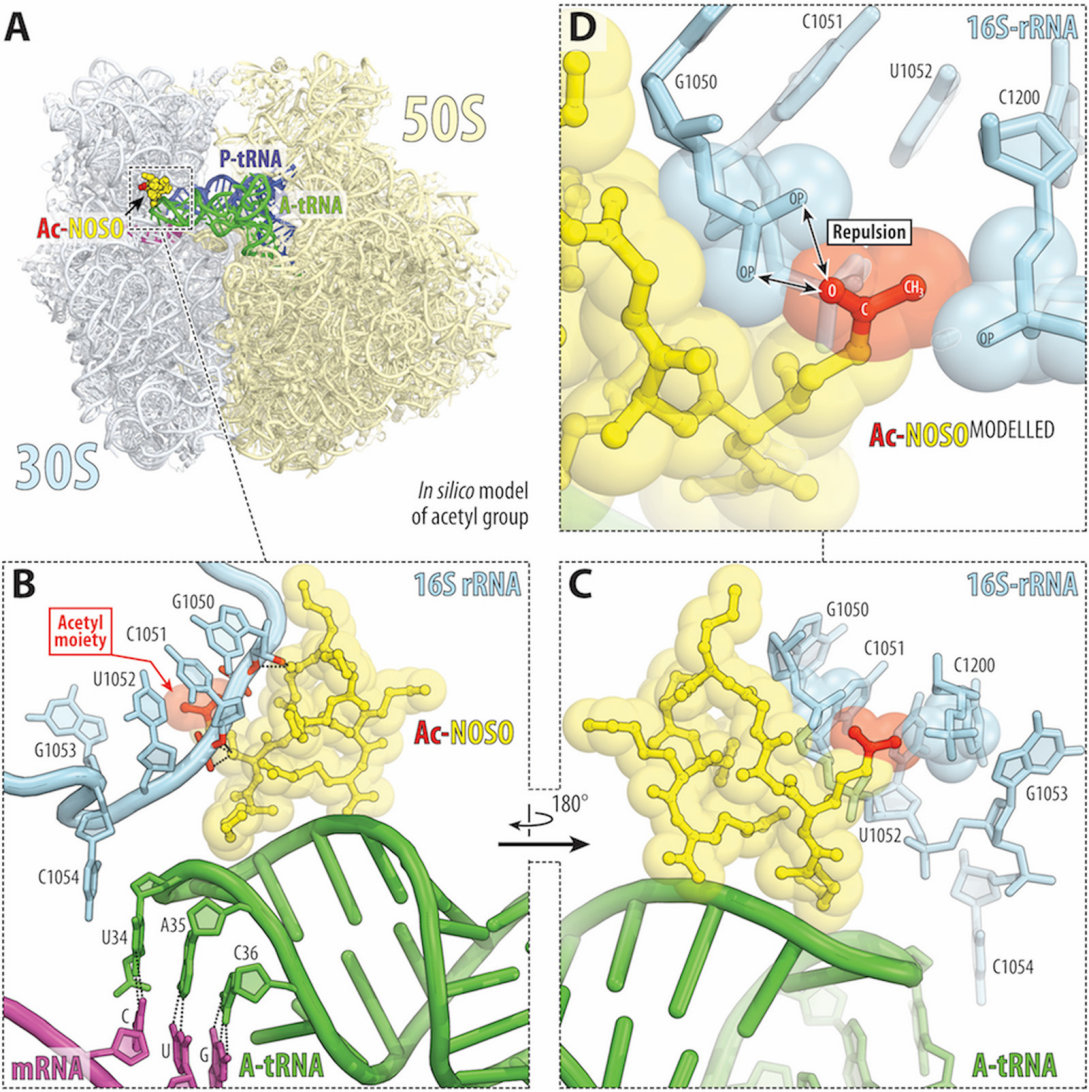
Structural basis for resistance to ODLs via acetylation by OatA enzyme. (A) Overview of the ODL drug-binding site (yellow) in the Thermus thermophilus 70S ribosome viewed as a cross-cut section through the nascent peptide exit tunnel. The 30S subunit is shown in light yellow, the 50S subunit is in light blue, the mRNA is in magenta, and the A- and P-site tRNAs are colored green and dark blue, respectively. The structure is from PDB entry 6CAE (18). (B to D) Close-up views of the *in silico*-modeled acetyl group (red) onto the side chain amino group of the Dab(βOH)_2_ of the ribosome-bound ODL (yellow) catalyzed by OatA. E. coli numbering of the 16S rRNA nucleotides is used. Note that the oxygen of the modeled acetyl group is placed next to the two negatively charged nonbridging oxygens of the C1051 phosphate, likely resulting in repulsion between them and decreased binding.

The binding sites of AGs and ODLs are adjacent to each other on the bacterial ribosome (18). It would, therefore, be interesting to compare the resistance strategies developed by bacteria against AGs with those against ODLs involving the expression of GNAT enzymes. AGs are broad-spectrum bactericidal compounds used to treat severe infections. For example, streptomycin, produced by Streptomyces griseus, was the first compound of this class to be identified. AGs contain typically one aminocyclitol ring linked via a glycosidic bond to several amino-modified sugars. AGs bind to helix 44 of the 16S rRNA in the 30S ribosomal subunit, causing translational errors and the inhibition of ribosome translocation. The *N*-acetylation of particular amino groups in AGs prevents the formation of some of the critical H-bonds between the drug and the 16S rRNA, resulting in a loss of antibacterial activity (36). The genes encoding aminoglycoside-modifying enzymes are generally carried by plasmids and transposons. It has been suggested that these enzymes originate from organisms producing aminoglycosides. In contrast, ODLs interact simultaneously with the 16S rRNA and the anticodon loop of the A-site aminoacyl-tRNA. Indeed, the α-amine of the Lys_1_ residue of the antibiotic forms an H-bond with the nonbridging phosphate oxygen of residue C32 in the anticodon loop of the A-site tRNA, whereas other ODL residues form multiple H-bonds with the residues of the 16S rRNA, in helices 31, 32, and 34 (18). Previous studies have shown that removal of Dab(βOH)_2_ or d-Orn_5_ from the ODL molecule strongly decreases both inhibitory capacity and the antibacterial activity of the altered compound (37). These data suggest that the amino group of the Dab(βOH)_2_ residue is of crucial importance for antibacterial activity. A high-resolution X-ray crystal structure of the bacterial 70S ribosome in complex with an ODL analog revealed that the side chains of Dab(βOH)_2_ and d-Orn_5_ interact directly with the sugar-phosphate backbone of nucleotides C1051 and C1214 of 16S rRNA helix 34, suggesting that they are likely to be important for the inhibition of translation (18). It is not, therefore, surprising that OatA confers resistance to ODLs predominantly by acetylating the side chain amino group of Dab(βOH)_2_, leading to a rapid loss of the translation-inhibiting and antibacterial properties of the compound. *In silico* modeling of the acetyl group on the side chain amino group of the Dab(βOH)_2_ of the ribosome-bound ODL (Fig. 4) suggested that the likely placement of the oxygen and the methyl of the acetyl group next to the negatively charged phosphates of residues C1051 and C1200, respectively, would result in repulsion and decreased binding of acetylated ODL to the ribosome, providing a possible structural basis for the poor antimicrobial activity of the acetylated compounds.

Interestingly, nonproteinogenic amino acids, such as Dab(βOH), have very rarely been described in previous studies (38), suggesting that OatA is selective. A similar observation was reported for edeines (EDEs), linear antibiotic pentapeptides produced by the soil bacterium Brevibacillus brevis. EDEs contain four nonproteinogenic amino acids. At high concentrations, they block bacterial translation by inhibiting the binding of fMet-tRNA to the P site of the 30S ribosomal subunit. EdeQ, an *N*-acetyltransferase, was shown to confer resistance to EDEs via the acetylation of a primary amino group of the unusual amino acid 2,3-diaminopropionic acid. The resulting *N*-acetylated compounds are unable to inhibit both translation *in vitro* and bacterial growth *in vivo* (7).

### *oatA* distribution, phylogenetic analysis, and linkage with the *odl* locus in the *Xenorhabdus* and *Photorhabdus* genomes.

A search of the NCBI database (15 July 2021) identified OatA orthologs (70% amino acid identity to OatA over at least 80% of the total length of the protein) only in phylum *Proteobacteria* and exclusively in a few *Xenorhabdus* and *Photorhabdus* species. Furthermore, no OatA orthologs were found when the MGnify database of the European Bioinformatics Institute (https://www.ebi.ac.uk/metagenomics/sequence-search/search/phmmer), which includes all types of microbiome-derived sequence data, was queried with the OatA_Xn_ sequence (15 July 2021).

**FIG 5:**
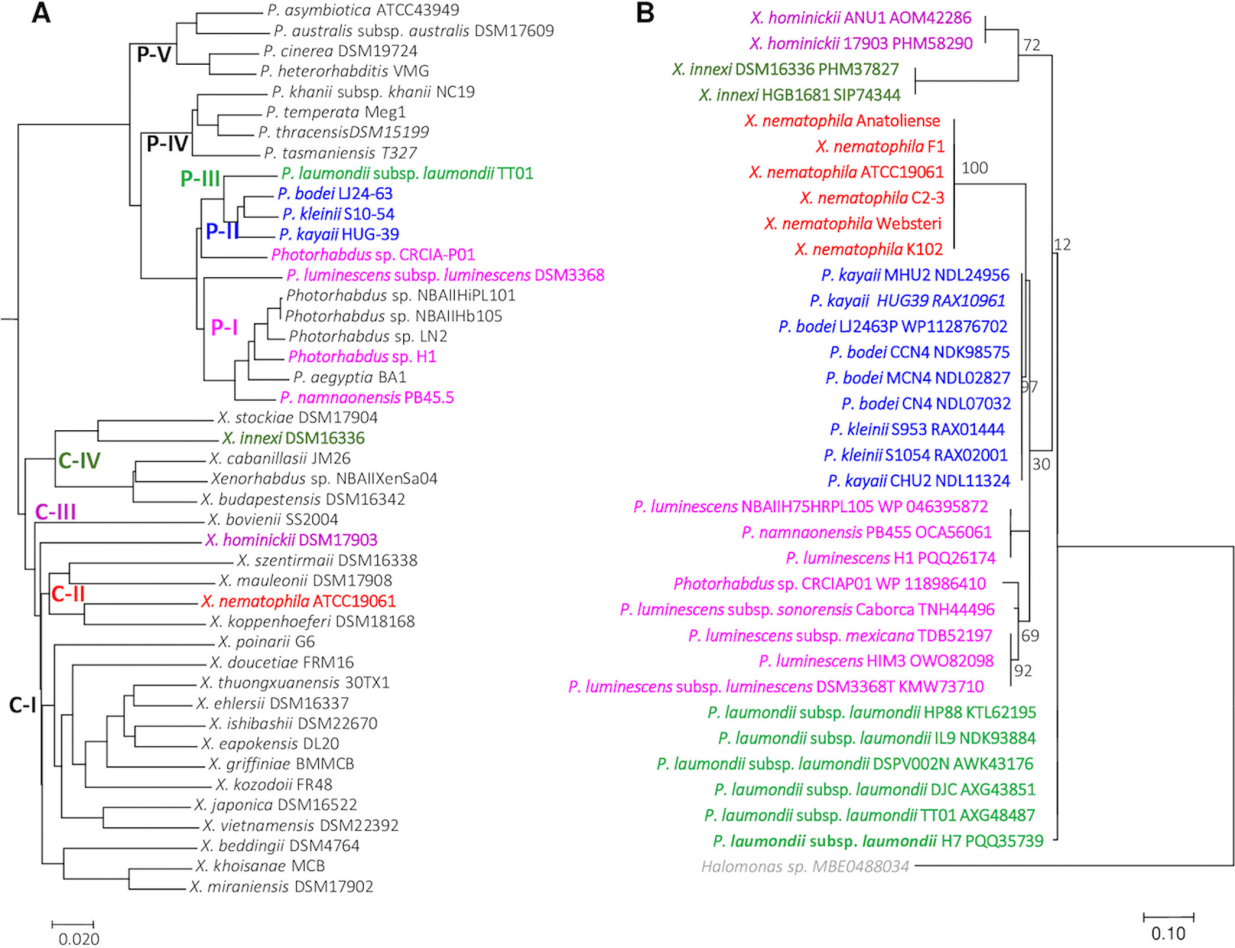
Distribution of acetyltransferase protein (OatA) orthologs among *Xenorhabdus* and *Photorhabdus* genomes. (A) Phylogenetic reconstruction based on 44 complete genome sequences of *Photorhabdus* and *Xenorhabdus* bacterial strains. Genomic similarity was estimated with Mash software (50), which calculates the distance between two genomes. This distance is correlated with average nucleotide identity (ANI) such that *D* = 1 − ANI. A tree was constructed dynamically from all the pairwise distances of the genome set, with the neighbor-joining JavaScript package. The clustering was computed from all-pair distances of ≤0.06 (≈94% ANI) corresponding to the standard ANI defining a species group. P-I to P-V are phylogenetic clades of *Photorhabdus* species; C-I to C-IV are phylogenetic clades of *Xenorhabdus* species. The names of the strains with *oatA* gene orthologs (see panel B) are shown in a particular color, depending on their species phylogenetic clade. (B) Phylogenetic analysis by the maximum likelihood method for the acetyltransferase protein (Oat). The orthologs of *X. nematophila* F1 OatA protein were identified by BLASTP analysis; only proteins with a sequence identity of ≥70% and minLrap of ≥80% were considered to be orthologs. The OatA sequence of *Halomonas* has been added as an outgroup sequence. The amino acid sequences were aligned by the Muscle algorithm, with the Seaview platform (51). Evolutionary analyses were inferred by the maximum likelihood method, based on the JTT matrix-based model (52), and were conducted in MEGA7 (53). The tree is drawn to scale, with branch lengths measured as the number of substitutions per site; bootstrap values are shown from 100 replicates. The analysis was performed on 35 amino acid sequences. All positions containing gaps and missing data were eliminated. In total, 132 positions were included in the final data set. The names of the strains are shown in a particular color according to the species phylogenetic clade (see panel A).

For the genus *Photorhabdus*, a molecular phylogenetic analysis (Fig. 5A) showed OatA to be present on several phylogenetic branches with a common ancestor corresponding to the former subspecies *P. luminescens* (39). OatA was, indeed, identified in clades P-I to P-III, which have now been raised to species level (*P. bodei*, *P. kayaii*, *P. laumondii*, and *P. luminescens*) (Fig. 5A) (40). The BLASTP and phylogenetic data confirm that OatA is absent from other major *Photorhabdus* clades, such as the P-IV clade, containing species with clinical strains (*P. asymbiotica* and *P. australis*) (Fig. 5). In the genus *Xenorhabdus*, OatA is found in only a few species spread over three clades (clades II, III, and IV) (Fig. 5).

**FIG 6:**
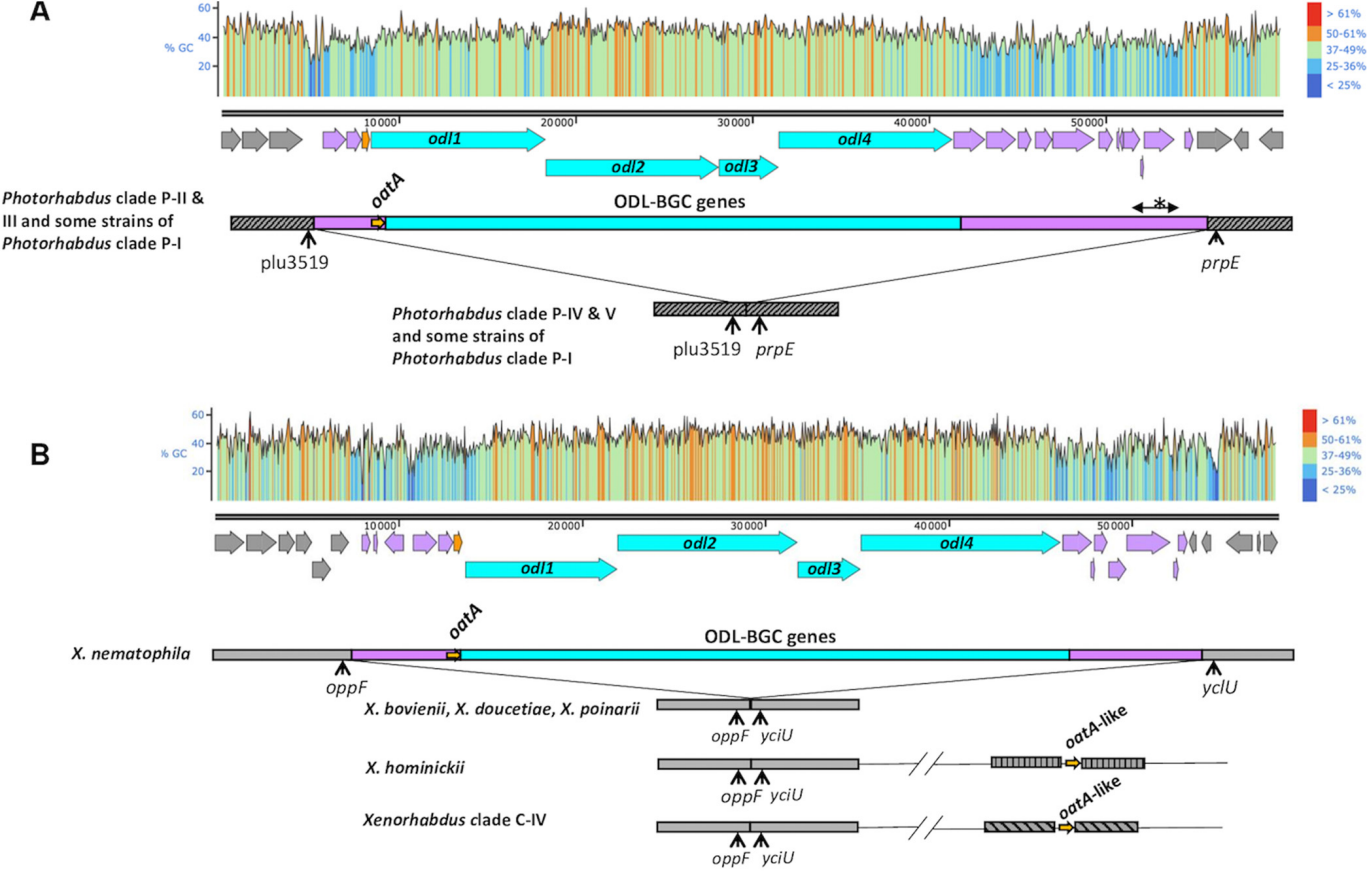
Genomic environment of the *oatA* gene in the *Photorhabdus* (A) and *Xenorhabdus* (B) genomes. Blue box, *odl*-BGC region; pink boxes, genomic regions framing the *odl*-BGC region encoding several enzymes, including OatA (the asterisk shows a highly shuffled subregion with a variable gene content according to the strain); gray boxes, core genome (different motifs indicate different regions of core genomes). GC content ({G or C}/{A, T, G, or C}) was calculated for a sliding window with a length of 25 nucleotides and plotted with SnapGene software (from Insightful Science). (A) Strains from clade P-I containing the *oatA* ortholog include *P. luminescens* subsp. *luminescens* DSM 3368, *P. luminescens* HIM3, *P. namnanoensis* PB45.5, and *Photorhabdus* sp. H1. Strains from the clade P-I which totally lack the *oatA* ortholog include *P. aegyptiae* BA1, *Photorhabdus* sp. LN2, NBAII HiPL 101, and NBAII Hb 105. Strains from clade P-II include *P. bodei* CN4 and LJ24-63, *P. kayaii* M-HU2, C-HU2, and HUG-39, *P. kleinii* S9-53 and S10-54, and *Photorhabdus* sp. CRCIAP01. Strains from clade P-III include *P. laumondii* TT01, HP88, and IL9. Strains from clade P-IV include *P. asymbiotica* ATCC 43949; *P. australis* subsp. *australis* DSM 17609, *P. cinnerea* DSM 19724, and *P. heterorhabditis* VMG. Strains from clade P-V include *P. temperata* J3, Meg1, and M1021, *P. thracensis* DSM 15199, *P. khani* NC19, and *P. tasmaniensis* DSM 22387. (B) *X. nematophila* strains include F1, ATCC 19061, K102 (CNCMI-4530), Anatoliense, Websteri, and C2-3. *X. hominickii* strains include ANU1 and DSM 17903. Strains from clade C-IV include *Xenorhabdus* sp. KK7.4 and KJ12.1, *X. innexi* HGB1681 and DSM 16336, *X. stockiae* DSM 17904, and *X. cabanillassii* 17905 and JM26. The *oatA* ortholog is totally absent from the strains of *Xenorhabdus* phylogenetic clades C-I and C-III, illustrated here by the strains *X. bovienii* CS03, *X. doucetiae* FRM16, and *X. poinarii* G6.

Given the large size of NRPS genes and their numerous intragenic repeats, reflecting the repetitive cycle of reactions catalyzed by enzymatic modules, we used DNA chips to obtain a complete picture of the occurrence profiles of the *odl*-BGC genes and their linkage to the *oatA* gene in strains from our laboratory collection of *Xenorhabdus* and *Photorhabdus* (57 and 20 strains, respectively) (41). The Agilent oligonucleotide microarray used in this study carries two or three 60-mer probes per sequence of interest. The 40 sequences of interest were either NRPS-PKS modules or nonmodular genes associated with NRPS-PKS loci identified in the genomic sequences of *X. nematophila* ATCC 19061^T^ and *P. laumondii* TT01^T^ (Fig. S7). We found that all *X. nematophila* strains, including the first ODL-producing strain ever described, K102, harbored the complete 44.5-kb locus carrying both the *odl*-BGC and the self-resistance gene, *oatA*. Complete *odl* loci, including *oatA* genes, were also present in *P. laumondii* and some *Photorhabdus* species (*P. luminescens*, *P. laumondii*, and *P. kayaii*). The *oatA* orthologs identified in *Photorhabdus* genomes flanked the *odl*-BGC locus (Fig. 6A). In contrast, within genus *Xenorhabdus*, this genomic organization was conserved only in the species *X. nematophila* (Fig. 6B). In both cases, the flanking regions of the *odl*-BGC locus have two remarkable features: their high content of enzyme-encoding genes, such as that encoding OatA, and their low GC content (Fig. 6). These features suggest that the large modular locus involved in ODL synthesis and self-resistance to ODLs was recently constructed by the addition of genomic modules in an as-yet-unidentified ancestor, followed by horizontal transfer to some *Photorhabdus* and *X. nematophila* species. Furthermore, the orphan *oatA* orthologs (i.e., *odl*-BGC independent) present in the genomes of Xenorhabdus hominickii and *Xenorhabdus* species from phylogenetic clade IV are located in radically different genomic environments; for this reason, they are considered to be *oatA*-like genes (Fig. 6B). Interestingly, the *oatA*-like orthologs of clade IV frequently present features of rearrangements likely to lead to pseudogenization (Fig. S8). These independent shuffling events in *oatA*-like genes strongly suggest the action of purifying selection in *Xenorhabdus*. Finally, a previous exhaustive description of regions of genomic plasticity (RGPs) in the genomes of *X. nematophila* ATCC 19061 and *P. laumondii* TT01 identified the *odl*-BGC locus as a mobile genetic element (MGE) (42). As described previously (42), such MGEs are composed of different genomic subregions, in this case the ODL synthesis and the ODL self-resistance subregions.

10.1128/mBio.02826-21.7 FIG S7 Oligonucleotide probe selection and odilorhabdin microarray synthesis. Two or three 60-mer oligonucleotide probes were designed by Imaxio (Biopôle Clermont-Limagne, Saint-Beauzire, France) for each NRPS-PKS genomic module or nonmodule gene of the *odl*-BGC from *X. nematophila* ATCC 19061 and *P. laumondii* TT01. The oligonucleotide probes have a mean *T_m_* of 80.37°C (±2.25°C), a mean GC of 43.33%, and no significant match elsewhere on the two bacterial genomes (less than 80% similarity over 30 nucleotides) to prevent cross-hybridization. Microarrays (8x15K) were synthesized with Agilent ink-jet technology (Agilent Technologies, Inc., Santa Clara, CA). Genomic DNA extraction, microarray hybridization, and microarray image analysis were done. We obtained bacterial genomic DNA for 77 strains from the DGIMI laboratory collection according to the method of Brenner et al. (2). For each genomic DNA, 0.5 μg of material was hydrolyzed with AluI and RsaI, denatured for 5 min at 95°C, and Cy3-labeled with a genomic DNA enzymatic labeling kit (Agilent Technologies) according to the manufacturer’s recommendations. Labeled targets were purified with Amicon 30-kDa columns (Millipore) and displayed a specific activity of 23 to 45 pmol of Cy3/μg. The Cy3-labeled genomic DNA was hybridized with the 60-mer oligonucleotide microarray at 65°C for 24 h. The arrays were scanned with Agilent Scanner (laser constant intensity) with the following settings: Dynamic AutoFocus, PMT Cy3 100%, 20 bit-data format, pixel resolution 5 μm. Microarray image files were extracted after intra-array normalization with Feature Extraction 10.7.3 software (Agilent Technologies). The mean background signal was calculated with the negative controls and subtracted from the mean signal for each spot. ODL biosynthesis gene distribution and profile clustering were done. After normalization and background correction, a sequence was considered present in the target genome if the hybridization signal intensity exceeded 1,000 for at least one of the probes. The hybridization profiles of each strain were transformed into binary numerical values (0, gene absent; 1, gene present). The dendrogram was generated by a hierarchical analysis of the distribution patterns (presence/absence) of the ODL-targeted probes according to the unweighted pair group method arithmetic mean (UPGMA) distance method and the Gower similarity coefficient (3), with Bionumerics software version 4.5 (Applied-Maths, Sint-Martens-Latem, Belgium). The taxonomic identities of the 77 strains used for hybridization experiment are shown on the right side of the dendrogram. The names of the NRPS-PKS genomic modules or nonmodule genes of the *odl*-BGC are shown at the top of the dendrogram. Download FIG S7, PDF file, 0.4 MB. Copyright © 2022 Lanois-Nouri et al. 2022 Lanois-Nouri et al. https://creativecommons.org/licenses/by/4.0/ This content is distributed under the terms of the Creative Commons Attribution 4.0 International license.

10.1128/mBio.02826-21.8 FIG S8 Pseudogenization of the *oatA*-like gene in the genomes of the strains from *Xenorhabdus* clade C-IV. Download FIG S8, PDF file, 0.1 MB. Copyright © 2022 Lanois-Nouri et al. 2022 Lanois-Nouri et al. https://creativecommons.org/licenses/by/4.0/ This content is distributed under the terms of the Creative Commons Attribution 4.0 International license.

### Intrinsic odilorhabdin resistance and OatA-dependent self-resistance in EPN-symbiotic bacteria.

We first investigated the functionality of *oatA*-like and *oatA* from *Xenorhabdus* and *Photorhabdus* by cloning and expressing these *oatA* genes in E. coli, as described above. As all *X. nematophila* OatA proteins have the same sequence, only the gene from the F1 strain was cloned. Table 3 clearly shows that the cloning and expression of the *oatA* genes from *Photorhabdus* and *X. nematophila* rendered E. coli resistant to NOSO-95C, with a MIC of at least 512 μg/mL. However, no significant resistance to NOSO-95C (Table 3) was observed after the cloning of *Xenorhabdus* orphan *oatA*-like genes displaying full sequence identity to *oatA-Xn* or truncated sequences (Fig. S8).

**TABLE 3:** MICs of NOSO-95C against E. coli recombinant strains expressing *oatA* or *oatA*-like genes and *X. nematophila* wild-type or mutant strains^*a*^

Type of strain	MIC (μg/mL) of NOSO-95C
E. coli expressing *oatA* from *Photorhabdus*	
XL1(P*_lac_*-*oatA-Pla-*HP88)	512
XL1(P*_lac_*-*oatA-Pla-*TT01)	512
XL1(P*_lac_*-*oatA-Pk*)	512
XL1(P*_lac_*-*oatA-Plu*)	1,024
E. coli expressing *oatA-like* from *Xenorhabdus*	
XL1(P*_lac_*-*oatA-like-Xc-*JM26)	8
XL1(P*_lac_*-*oatA-like-Xc-*DSM 17905)	8
XL1(P*_lac_*-*oatA-like-Xh*)	32
XL1(P*_lac_*-*oatA-like-Xi*)	4
XL1(P*_lac_*-*oatA-like-Xs*)	8
Controls	
XL1 (pUC19)	8
XL1(P*_lac_*-*oatA-Xn*)	1,024
*X. nematophila* strain F1	
Wild type	>2,048
Mutant strain (Δ*ngrA*)	>2,048
Double mutant strain (Δ*ngrA* Δ*oatA*)	256

a *Pla*, *P. laumondii*; *Pk*, *P. kayaii* KR04; *Plu*, *P. luminescens* Hb; *Xc*, *X. cabanillasii*; *Xh*, *X. hominickii* DSM 17903; *Xi*, *X. innexi* UY61; *Xs*, *X. stockiae* DSM 17904.

We needed an *oatA* mutant for assessment of the relative contribution of OatA to overall ODL resistance. We constructed this mutant in an *ngrA*-deficient strain of *X. nematophila*, to prevent the suicide of the producing strain. A comparison of the MICs of NOSO-95C against *X. nematophila* (*ngrA* mutant) and its derivative *oatA* strain revealed a decrease in resistance by a factor of more than 8. However, a high MIC (256 μg/mL) was still observed for the *oatA* mutant (Table 3), suggesting the existence of another intrinsic resistance mechanism in *X. nematophila*. We therefore evaluated ODL resistance in a large collection of EPN-symbiotic bacteria. The ODL resistance profiles of *Photorhabdus* strains were simple. Basically, only strains from clades I to III (Table 4) with both the *odl*-BGC locus and an *oatA* self-resistance gene were resistant to ODLs, with an MIC of >512 μg/mL. For *X. nematophila*, the first ODL-producing strain identified, *X. nematophila* K102 (18), and the other *X. nematophila* strains displayed high-level resistance to NOSO-95C (Table 4). This resistance was associated with the presence of *oatA* in the genome (Fig. S7), confirming that *X. nematophila* has developed a mechanism of self-resistance to ODLs. However, other *Xenorhabdus* species displayed various degrees of susceptibility to the natural compound. For the most susceptible strains (Table 4), MICs ranged from 8 to 128 μg/mL. In contrast, the strains of several other species were highly resistant despite the absence of an *oatA* gene (e.g., *X. koppenhoeferi* DSM 18168) (Fig. S7) or the presence of a nonfunctional *oatA*-like resistance gene (like *X. innexi* DSM 16336) (Fig. S8). As-yet-unexplained OatA-independent resistance phenomena also occur in M. morganii (Table 1). Our findings also indicate that there is no correlation between intrinsic resistance to ODLs and taxonomic clade in *Xenorhabdus* (Table 4).

**TABLE 4:** MICs of NOSO-95C against *Xenorhabdus* and *Photorhabdus* strains

Strain	Clade	MIC (μg/mL) of NOSO-95C
*Xenorhabdus* species		
Xenorhabdus bovienii DSM 4766	*Xenorhabdus* clade (C-III)	16
Xenorhabdus cabanillasii DSM 17905	*Xenorhabdus* clade (C-IV)	1,024
Xenorhabdus doucetiae FRM16	*Xenorhabdus* clade (C-I)	1,024
Xenorhabdus griffiniae DSM 17911	*Xenorhabdus* clade (C-I)	32
Xenorhabdus hominickii DSM 17903	*Xenorhabdus* clade (C-II)	32
Xenorhabdus indica DSM 17382	*Xenorhabdus* clade (C-IV)	128
Xenorhabdus innexi UY61^*a*^	*Xenorhabdus* clade (C-IV)	1,024
Xenorhabdus ishibashii DSM 22670	*Xenorhabdus* clade (C-I)	8
Xenorhabdus koppenhoeferi DSM 18168	*Xenorhabdus* clade (C-II)	>512
Xenorhabdus mauleonii DSM 17908	*Xenorhabdus* clade (C-II)	256
Xenorhabdus nematophila DSM 3370^T^	*Xenorhabdus* clade (C-II)	>512
Xenorhabdus nematophila F1^*a*^	*Xenorhabdus* clade (C-II)	>2,048
Xenorhabdus nematophila CNCM I-4530 (K102)^*a*^	*Xenorhabdus* clade (C-II)	>2,048
Xenorhabdus poinarii DSM 4768	*Xenorhabdus* clade (C-I)	16
Xenorhabdus romanii DSM 17910	*Xenorhabdus* clade (C-I)	32
Xenorhabdus szentirmaii DSM 16338	*Xenorhabdus* clade (C-II)	32
*Photorhabdus* species		
*Photorhabdus laumondii* TT01^*a*^	*Photorhabdus* clade (P-III)	512
Photorhabdus luminescens Hb^*a*^	*Photorhabdus* clade (P-I)	2,048
*Photorhabdus kayaii* KR04^*a*^	*Photorhabdus* clade (P-II)	512
Photorhabdus asymbiotica ATCC 43949	*Photorhabdus* clade (P-V)	64
Photorhabdus tasmaniensis NZ48^*a*^	*Photorhabdus* clade (P-IV)	64
Photorhabdus temperata XlNach^*a*^	*Photorhabdus* clade (P-IV)	16

a From DGIMI laboratory collection.

Our study clearly reveals that the resistance gene, *oatA*, is found exclusively in EPN-symbiotic bacteria. It is therefore confined to EPN-infected insects and IJs harboring specific bacterial symbiont species. However, bacterial symbionts can persist for several weeks in the cadaver of the insect before reassociating with new generations of IJs, and it has been suggested that they need to produce broad-spectrum antimicrobial compounds to protect the insect cadaver from competing soil microorganisms (43). This raises the question of whether the ODLs exert selection pressure in EPN-infested cadavers, favoring the HGT of the *oatA* self-resistance gene to bacteria with no intrinsic ODL production capacity. It has been shown in bacterial competition experiments that Staphylococcus saprophyticus is eliminated following its introduction into growing cultures *in vitro* (mimicking the hemolymph) of either the xenocoumacin mutant strain or the ODL mutant strain but that this bacterium is able to grow in the presence of the *ngrA* strain, indicating that the main water-soluble antimicrobial agent, xenocoumacin, and ODLs were not required to eliminate the competitor. In addition, natural infection experiments with insect larvae have shown that NgrA-independent antibiotics are involved in *S. saprophyticus* control. Finally, it has been shown that NgrA-derived compounds from *X. nematophila* serve as signals for nematode reproduction *in vivo* (30).

In response to the urgent need to address the lack of effective treatments for the increasing number of human infections caused by Gram-negative multidrug-resistant bacteria, ODLs, a promising new family of antibiotics produced by *X. nematophila*, are currently in preclinical development. EPN-symbiotic bacteria have developed a successful strategy to avoid being killed by their own antibiotic products. OatA, a GNAT encoded by the *odl*-BGC, confers selective resistance to ODLs by predominantly acetylating the side chain amino group of one of its rare amino acids, leading to a rapid loss of the ability to inhibit translation and the antibacterial properties of these compounds. A link has been established between antibiotic production by bacteria residing in environments not significantly affected by human antibiotic use and antimicrobial resistance and was first reported by Benveniste and Davies in 1973 (44). We show here that ODL resistance genes are present only in bacterial species belonging to the same phylogenetic cluster as the producing strain, which is specific to a particular niche, soil-dwelling EPNs. In addition, no trace of *oatA* HGT was detected, excluding the presence of this gene in the principal bacteria pathogenic to humans. This is an important point in the development of a new antibiotic. This work thus provides a perfect example of the coevolution of antibiotic production and self-resistance mechanisms in Gram-negative bacteria.

## MATERIALS AND METHODS

### Bacterial strains, plasmids, and growth conditions.

The strains and plasmids used in this study are listed in Table S1. Bacteria were routinely grown in Luria-Bertani (LB) medium at 28°C (*X. nematophila*) or 37°C (E. coli). When required, antibiotics were used at the following final concentrations: kanamycin, 20 μg/mL (or 10 μg/mL for chromosome selection); gentamicin, 15 μg/mL; chloramphenicol, 20 μg/mL for E. coli strains and 15 μg/mL for *X. nematophila* (or 8 μg/mL for chromosome selection); and ampicillin, 100 μg/mL. P*_tet_* constructs were induced by adding anhydrotetracycline (aTc) at a final concentration of 0.2 μg/mL. The P*_ara_* ETγA (full-length *recE*, *recT*, *redγ*, and *recA*) and P*_ara_* γβαA (*redγ*, *redβ*, *redα*, and *recA*) operons were induced by adding 10% l-arabinose. P*_lac_* constructs were induced by adding IPTG at a final concentration of 0.2 mM. We added 100 μg/mL of diaminopimelic acid (DAP) to cultures of the E. coli WM3064 strain, which is auxotrophic for this amino acid.

10.1128/mBio.02826-21.9 TABLE S1 Strains and plasmids used in this study. Download Table S1, PDF file, 0.1 MB. Copyright © 2022 Lanois-Nouri et al. 2022 Lanois-Nouri et al. https://creativecommons.org/licenses/by/4.0/ This content is distributed under the terms of the Creative Commons Attribution 4.0 International license.

### Molecular genetic techniques.

DNA manipulations were performed as previously described (45). Plasmids were introduced into E. coli by transformation and transferred to *X. nematophila* by conjugative mating with WM3064 as the donor strain (46). All constructs were sequenced by Eurofins Genomics Germany GmbH. The primers used in this study (IDT) are described in Table S2.

10.1128/mBio.02826-21.10 TABLE S2 Primers used in this study. Download Table S2, PDF file, 0.1 MB. Copyright © 2022 Lanois-Nouri et al. 2022 Lanois-Nouri et al. https://creativecommons.org/licenses/by/4.0/ This content is distributed under the terms of the Creative Commons Attribution 4.0 International license.

### Recombineering construction of p15A-P*_tet_-odl*-BGC-mob.

As previously described by Fu et al. (29), genomic DNA from *X. nematophila* was first digested with NcoI and SphI to generate a 45.2-kb fragment containing the *odl*-BGC locus and surrounding genes. Homology arms were ordered as oligonucleotides and attached by p15A-cm PCR with pACYC184 as a template. One homology arm started at the start codon of the first gene cloned (*ectB* gene) and extended 80 bp downstream. The other homology arm started at the SphI site and extended 80 bp upstream. After linear-plus-linear homologous recombination by RecET in GB05-dir activated with arabinose (0.3%), the *odl* locus was inserted directly into the p15A vector to create the p15A-*odl*-BGC containing a 44.5-kb *odl*-BGC fragment (four *odl* genes plus the surrounding genes). For expression of the *odl*-BGC locus, homology arms were ordered as oligonucleotides and attached to the Km^r^-Tet^r^-P_LtetO1_ fragment of pSC101. One homology arm started at the start codon of the *ectB* gene and extended 57 bp downstream. The other homology arm started 216 bases from the p15A origin and extended 52 bp upstream. After linear-plus-circular homologous recombination by Redαβ in GB08-red activated with arabinose (0.3%), the Km^r^-Tet^r^-P_LtetO1_ fragment was inserted into the p15A-*odl*-BGC plasmid between the p15A origin and the start codon of the *ectB* gene, to create p15A- P*_tet_-odl*-BGC containing the 44.5-kb *odl*-BGC fragment under the control of the P_LtetO1_ promoter. For complementation of the *odl* mutant with p15A-P*_tet_-odl*-BGC, homology arms were ordered as oligonucleotides and attached to the Gm^r^-*mob* fragment of pBBR1-MCS5. One homology arm started at the end of the kanamycin resistance gene sequence and extended 57 bp downstream. The other homology arm started at the end of Tet^r^ and extended 54 bp upstream. After linear-plus-circular homologous recombination by Redαβ in GB08-red activated with arabinose (0.3%), the Gm^r^-*mob* fragment was inserted directly into the p15A-P*_tet_-odl*-BGC plasmid, between Km^r^ and Tet^r^, to create the mobilizable plasmid p15A-P*_tet_*-*odl*-BGC-mob.

### Insertion of diverse genes into E. coli XL1 Blue MRF′.

For identification of the odilorhabdin self-resistance gene, the regions upstream and downstream from the *odl* locus of *X. nematophila* F1 were amplified by PCR with the L-*ectB*-KpnI and R-c-SacI primers (for *abc* genes) or the L-d-KpnI and R-j-SacI primers (for *defghij* genes), respectively, and inserted into pUC19 digested with KpnI and SacI. Genes *a*, *b*, and *c* were amplified separately, by PCR, with L-*ectB*-KpnI and R-*ectB*-SacI, L-b-KpnI and R-b-SacI, and L-c-KpnI and R-c-SacI, respectively, and inserted into pUC19 digested with KpnI and SacI. All these constructs were used to transform E. coli XL1-Blue MRF′ and were assessed separately in odilorhabdin MIC assays after the addition of IPTG. We assessed the functionality of diverse OatA proteins from *Photorhabdus* strains or OatA-like proteins from *Xenorhabdus* strains, by amplifying the corresponding genes by PCR with appropriate primers (Table S2) and inserting the amplicon between the KpnI and SacI sites of pUC19. The resulting constructs were then used to transform the E. coli XL1 Blue MRF′ strain, and odilorhabdin MIC assays were performed after adding IPTG. The *ngrA* PCR fragment was inserted into the pBBR1-MCS2 broad-host-range vector with the L-ngrA-EcoRI and R-ngrA-BamHI primers digested with EcoRI and BamHI, and the resulting construct was used to transform E. coli XL1 Blue MRF′ before the addition of p15A-P*_tet_*-*odl*-BGC.

### Cloning, overexpression, and purification of OatA.

For purification of the *N*-acetyltransferase, the *oatA* gene was first amplified from *X. nematophila* genomic DNA with the F-Nterm-oatA-NdeI and R-Stop-oatA-SalI primers (Table S2), digested with NdeI and SalI, and inserted into pET-28a previously digested with the same enzymes, at a position downstream from the T7 promoter and in fusion with the His tag, yielding pET-*oatA*. Fresh precultures of the overexpressing strain BL21(DE3) harboring the pET-*oatA* plasmid were grown overnight in 20 mL of LB broth supplemented with 20 μg/mL of kanamycin at 35°C. We inoculated 200 mL of LB broth supplemented with 20 μg/mL of kanamycin in a 1-L Erlenmeyer flask with fresh overnight preculture (2% [vol/vol]) and incubated it at 35°C with shaking until the culture reached an optical density at 600 nm (OD_600_) of 0.4. The cells were induced by incubation with 0.2 mM IPTG for 3 h at 35°C. Cell pellets from two flasks were recovered by centrifugation at 6,000 × *g* for 10 min, pooled, and washed once with saline. The resulting cell pellet was resuspended in 4 mL of 50 mM HEPES buffer (pH 7.5), 100 mM NaCl, and 2% glycerol (ACT buffer). Cells were lysed by processing the cell suspension in a Fast-Prep-24 instrument (MP Biomedicals) for 20 s at a speed of 4 m/s with 100-μm glass beads/cell at a suspension ratio of 1/3. The samples were then centrifuged (13,000 × *g* for 5 min) and the supernatants were recovered. Cell pellets were washed once with ACT buffer and the supernatants were pooled. Imidazole was added to a concentration of 10 mM before enzyme purification by the HisPur nickel-nitrilotriacetic acid (Ni-NTA) Superflow agarose batch method (Thermo Fisher Scientific). Briefly, 1 mL of equilibrated Ni-NTA resin was added to 3 mL of sample. The mixture was incubated for 1 h with gentle shaking and was then washed four times with 25 mM imidazole in ACT wash buffer. Three elutions were performed, with 150, 250, and 500 mM imidazole. The eluates obtained with 250 and 500 mM imidazole were pooled and subjected to dialysis overnight against 75 mM HEPES at 4°C with a PurA-Lyzer cutoff at 3.5 kDa. Glycerol was added to the purified His-tagged OatA enzyme at a final concentration of 10% before storage at −20°C.

### Production and LC-MS analysis of secondary metabolites.

For the detection of secondary metabolites in the culture supernatants, bacteria were collected from 96-h cultures by centrifugation at 7,000 × *g* for 10 min and the supernatants after filtration on 0.2-μm filters were analyzed by LC-MS on an Agilent 1260 Infinity system coupled to an Agilent 6120 quadrupole mass spectrometer. The conditions for high-performance liquid chromatography (HPLC) separation were as follows: a Symmetry C_18_ analytical column (5 μm, 4.6 mm by 150 mm; Waters Corporation) was used in positive mode, the injection volume was 100 μL, mobile phase A was 0.1% trifluoroacetic acid in water, mobile phase B was acetonitrile, and the linear gradient was 0% mobile phase B–100% mobile phase A at 0 min to 80% mobile phase B–20% mobile phase A at 40 min. UV detection was performed at 210 nm. During separation, the flow rate was 0.7 mL/min, and the column temperature was 37°C.

### *In vitro* acetylation of NOSO-95C.

*N*-Acetylation of NOSO-95C or NOSO-95179 was performed in a polystyrene microtube with purified His-tagged OatA diluted to 300 μg/mL in 75 mM HEPES (pH 7.5) supplemented with glycerol (5.7% [vol/vol]), 250 μM NOSO-95C or NOSO-95179, and 750 μM acetyl-CoA in 75 mM HEPES (pH 7.5) for 24 h at 28°C. Aliquots were sampled at 15 min, 4 h, and 24 h.

### LC-MS/MS analysis.

The acetylated NOSO-95C compounds were analyzed by LC-MS/MS. Data were obtained in the positive mode on a Waters Alliance LC-MS/MS system (Waters Synapt G2-S mass spectrometer, Waters UPLC Acquity H-class). The HPLC column used was a Phenomenex EVO C18 T HPLC column (100 by 2.1 mm, 1.7 μm) maintained at 25°C. Solvent A was H_2_O plus 0.1% (vol/vol) formic acid, solvent B was acetonitrile plus 0.1% (vol/vol) formic acid, and the flow rate was 0.5 mL/min. The mobile phase composition was 100% solvent A from 0 min to 5 min, ramped to 30% solvent B at 10 min. Sample injection volume was 0.1 μL. UV-visible detection was by absorbance at 200 to 500 nm. An ion spray (IS) voltage of 3,000 V, a source temperature of 140°C, a desolvation temperature of 450°C, a declustering potential (DP) of 30 V, and a collision energy (CE) in the range of 20 to 60 V were applied.

### Antibiotic activity.

Total antimicrobial activity was assessed with Klebsiella pneumoniae ATCC 43816 as a target of strains harboring *odl*-BGC loci. We placed 15 μL of an overnight bacterial culture on a plate containing Mueller-Hinton broth (MHB; Bio-Rad) supplemented with 1.4% agarose (Sigma), and aTc (200 ng/mL) was added when required. After incubation for 72 h at 28°C, the bacteria were exposed to chloroform fumes for 30 min and allowed to dry in air for 30 min. The Klebsiella pneumoniae ATCC 43816 (200 μL of an overnight culture) target strain was then added to 8 mL of MHB supplemented with 0.7% agarose, which was poured over the bacterial colony. After incubation for 24 h at 37°C, zones of growth inhibition were observed. The antibiotic activity of the samples acetylated *in vitro* was also assessed, as follows. We spotted 10 μL of sample onto Mueller-Hinton II (Becton, Dickinson) medium supplemented with 1.4% agarose. We poured an overnight culture of E. coli ATCC 25922 in LB broth (2% [vol/vol]) over the plate. The plate was allowed to dry and was then incubated at 35°C for 20 h. We then observed the plates to check for zones of growth inhibition.

### IVTT assay.

The effect of *in vitro* acetylation on bacterial protein synthesis was investigated in the Expressway cell-free E. coli expression system (Invitrogen). The green fluorescent protein (GFP) gene was amplified from the pCmGFP plasmid (47) and inserted into pEXP5-CT TOPO (Thermo Fisher Scientific), and the resulting plasmid was used as a template for *in vitro* transcription and translation (IVTT). Reactions were performed according to the manufacturer’s protocol, in 50 μL of reaction mixture, in the wells of a black polystyrene 96-half-well microplate (Greiner). Feed buffer was added after 30 min of incubation, to ensure optimal protein synthesis. Reactions were initiated by adding 1 μg of plasmid DNA. The plate was incubated at 30°C and fluorescence was measured every 20 min (λ_ex_ = 475 nm; λ_em_ = 520 nm) with a Spark M10 microplate reader (Tecan). Fluorescence from GFP synthesis was recorded at 20 min after addition of feed buffer using GraphPad Prism 6 software. Gentamicin was used as positive control at 10 μM.

### Gene expression and identification of the compounds produced in complemented strains.

We investigated the production of secondary metabolites in complemented strains, by incubating 100-mL broth cultures of Δ*xcnKL*::*odl1* (MCS5) and Δ*xcnKL*::*odl1* (p15A-P*_tet_*-*odl*-BGC-mob) strains with gentamicin and aTc at 28°C for 78 h, centrifuging the cultures, and passing the supernatants through 0.2-μm filters and concentrating them 10-fold for LC-MS analysis. Natural odilorhabdins (NOSO-95A, -B, and -C) and xenocoumacin 1 (Xcn 1) were identified in culture supernatants by LC-MS on an Agilent 1260 Infinity system coupled to an Agilent 6120 quadrupole mass spectrometer. The conditions for HPLC separation were as follows: a Symmetry C_18_ analytical column (5 μm, 4.6 mm by 150 mm; Waters Corporation) was used in positive mode, the injection volume was 100 μL, mobile phase A was 0.1% trifluoroacetic acid in water, mobile phase B was acetonitrile, and the linear gradient was 0% mobile phase B–100% mobile phase A at 0 min to 80% mobile phase B–20% mobile phase A at 40 min. UV detection was performed at 210 nm. During separation, the flow rate was 0.7 mL/min, and the column temperature was 37°C.

### Construction of *X. nematophila* mutants.

For the construction of a Δ*oatA* mutant, we first constructed an *ngrA*-deficient strain, to prevent the suicide of the *X. nematophila* producer strain. The upstream and downstream regions of the *ngrA* gene were amplified by PCR with the L-PCR1-ngrA-XbaI and R-PCR1-ngrA-BamHI primers for the upstream region (579 bp) and the L-ngrA2bis-BamHI and R-ngrA2bis-ApaI primers for the downstream region (522 bp). The two fragments obtained were inserted, together with the 3.8-kb BamHI fragment omega interposon cassette from pHP45-ΩCm conferring resistance to chloramphenicol, into pJQ200SK digested with XbaI and ApaI for insertion of the ΩCam cassette between the two PCR fragments. The resulting plasmid, pJQ-*ngrA*::ΩCm, was used to transform E. coli strain WM3064 and was introduced into *X. nematophila* F1 by mating. Allelic exchange was performed as previously described (48). Omega insertion was confirmed by PCR analysis, and the loss of NRP products was checked by antibiosis tests. The clone obtained was called the Δ*ngrA* clone. For construction of the Δ*ngrA* Δ*oatA* double mutant, the upstream and downstream regions of the *oatA* gene were amplified by PCR with the appropriate primer pairs, L-SacI-b/R-SpeI-oatA (upstream) and L-SalI-oatA/R-ApaI-odl1 (downstream), generating a 595-bp PCR1 fragment and a 611-bp PCR2 fragment, respectively (Table S2). The pJQ-KmT1 plasmid was then digested with SpeI and SalI and the 2-kb KmT1 fragment containing the Km^r^ cassette and T1 terminators was purified with the Zymo gel purification kit (Ozyme). These three fragments were inserted into pJQ200SK digested with SacI and ApaI to introduce the Km-T1 cassette between the two PCR fragments. The pJQ-*oatA*::KmT1 plasmid was then introduced, by mating, into the *X. nematophila ngrA* mutant, and allelic exchange was performed. Km^r^ and Sac^r^ exconjugants were selected on LB agar supplemented with 4% sucrose and kanamycin. The KmT1 insertion was checked by PCR analysis with the two primers L-b-KpnI/R-Km-PvuI amplifying a 1,791-bp fragment (Table S2), with confirmation by DNA sequencing (Eurofins Genomics). The resulting recombinant clone was named the Δ*ngrA* Δ*oatA* clone.

### MIC determination.

MICs were determined in triplicate, in accordance with CLSI guidelines but with modifications for *Xenorhabdus* and *Photorhabdus* strains and for E. coli constructs (49). For *Xenorhabdus* and *Photorhabdus* strains, inoculum preparation was modified as follows: 3 mL of MHB II (Becton, Dickinson, Thermo Fisher Scientific) was inoculated with bacteria grown overnight in LB broth and cultured to an OD_600_ of 0.11 to 0.15. The inoculum was prepared as described in the CLSI guide, with a 1:20 dilution in saline. Multiplates were incubated for 24 h at 28°C or for longer if the results were unclear. For E. coli constructs, a modified method of inoculum preparation was used in which about 10 colonies were picked from MH agar supplemented with 100 μg/mL of ampicillin and used to inoculate 10 mL of MHB II supplemented with 100 μg/mL of ampicillin. The inoculum was incubated for 1.5 to 2 h at 35°C, with shaking at 150 rpm, until the OD_600_ reached 0.15 to 0.25. IPTG was added at a concentration of 0.2 mM for induction, and the culture was incubated for 30 min at 35°C, with shaking at 150 rpm. The inoculum was then diluted 1:20 in saline to obtain the final inoculum. The reaction medium consisted of MHB II supplemented with 0.2 mM IPTG. For other E. coli constructs requiring aTc induction, aTc was added to the isolate used for streaking and the reaction medium at a concentration of 200 ng/mL. In all other respects, the procedure corresponded to the CLSI method.
