# Supplementary material for: The Odilorhabdin Antibiotic Biosynthetic Cluster and Acetyltransferase Self-Resistance Locus Are Niche and Species Specific
Source: mBio. 2022 Jan 11;13(1):e02826-21. doi: 10.1128/mbio.02826-21 (PMC8749412; doi:10.1128/mbio.02826-21)

Fig. S1. LC-MS analysis of *X. nematophila* WT, mutants and complemented strains for the production of odilorhabdins (NOSO-95A, B and C) and Xenocoumacin. Supernatants from 96h-cultures (or 78h-cultures from complemented strains) were filtered onto 0.2µm and analyzed as previously described in Methods by LC-MS at 210 nm with molecular masses from 630 to 650 (for ODLs) and from 465 to 467 (for Xcn 1) after extracted ion chromatographic (EIC) analysis.

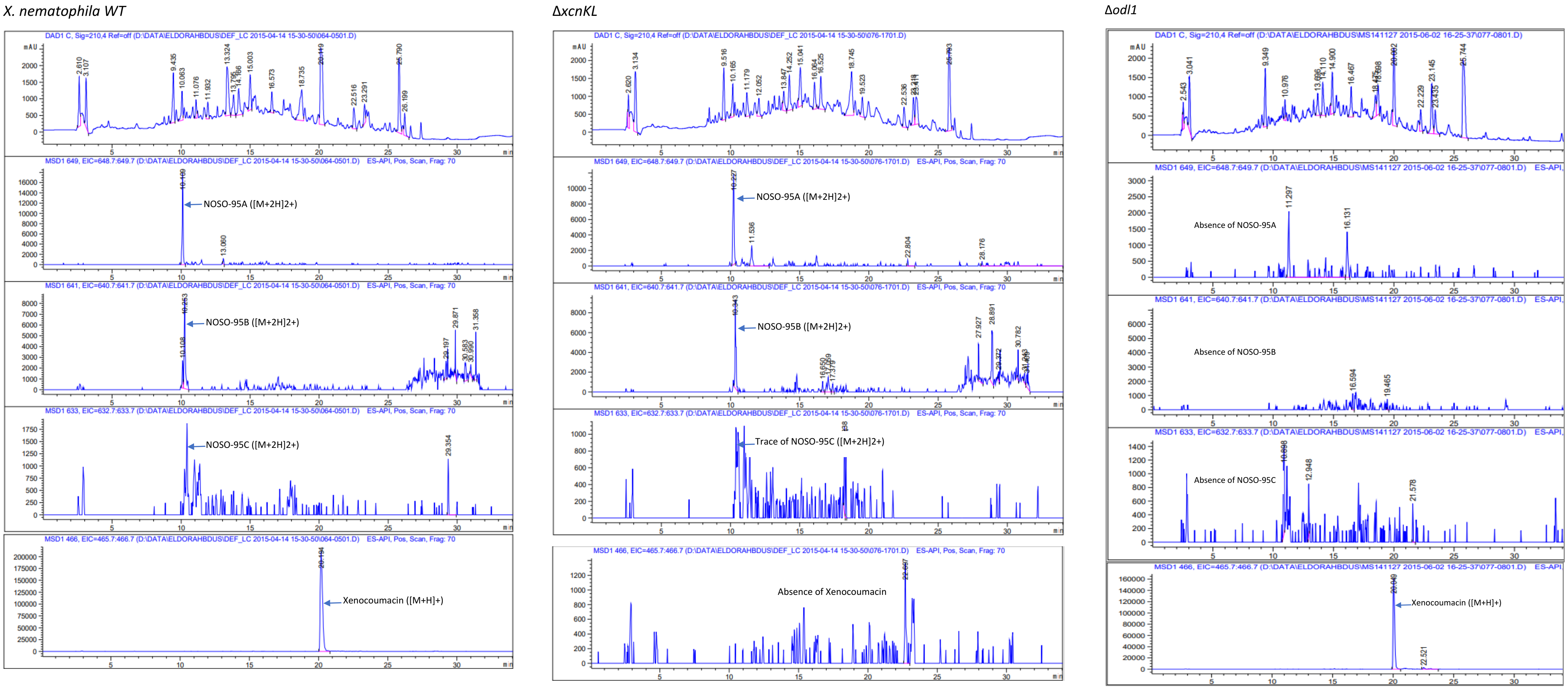

Δ*xcnKL* ::*odl1*

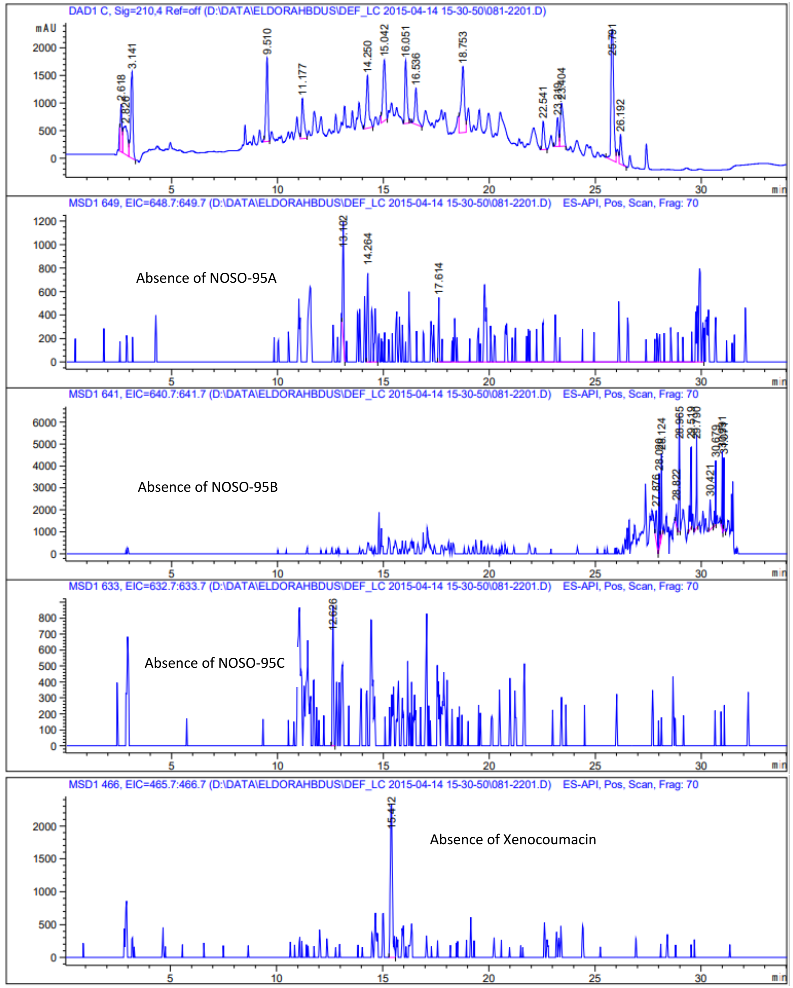

Δ*xcnKL* ::*odl1* (pBBR1-MCS5) + aTc

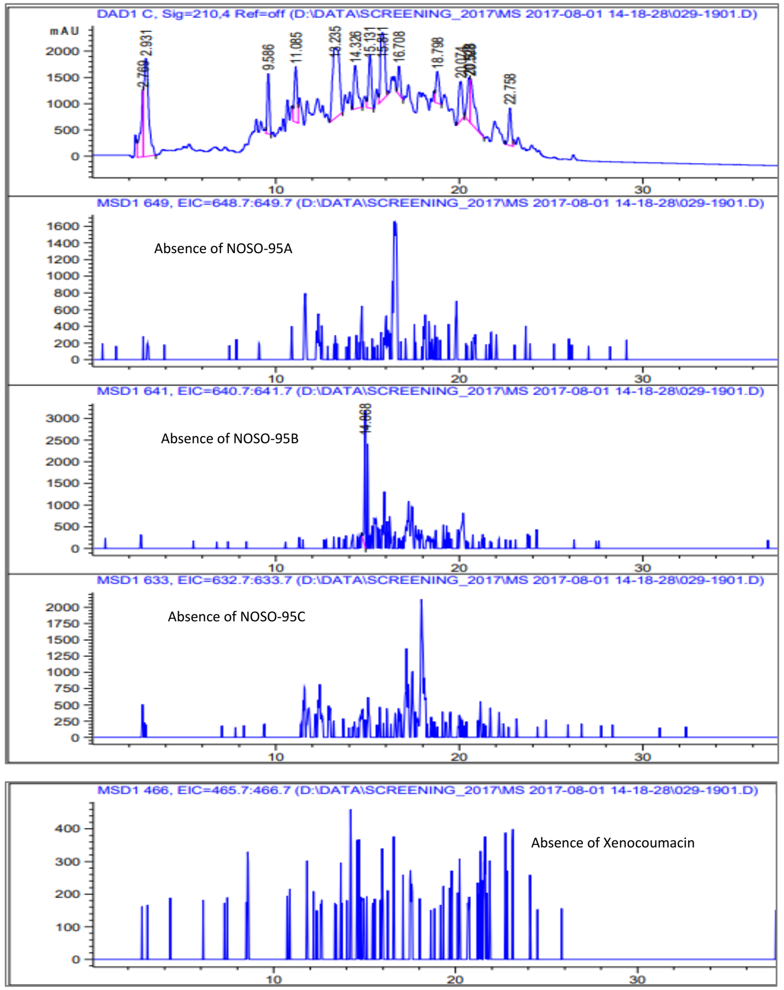

Δ*xcnKL* ::*odl1* (p15A-P$_{tet}$-*odl*-BGC-mob) +aTc

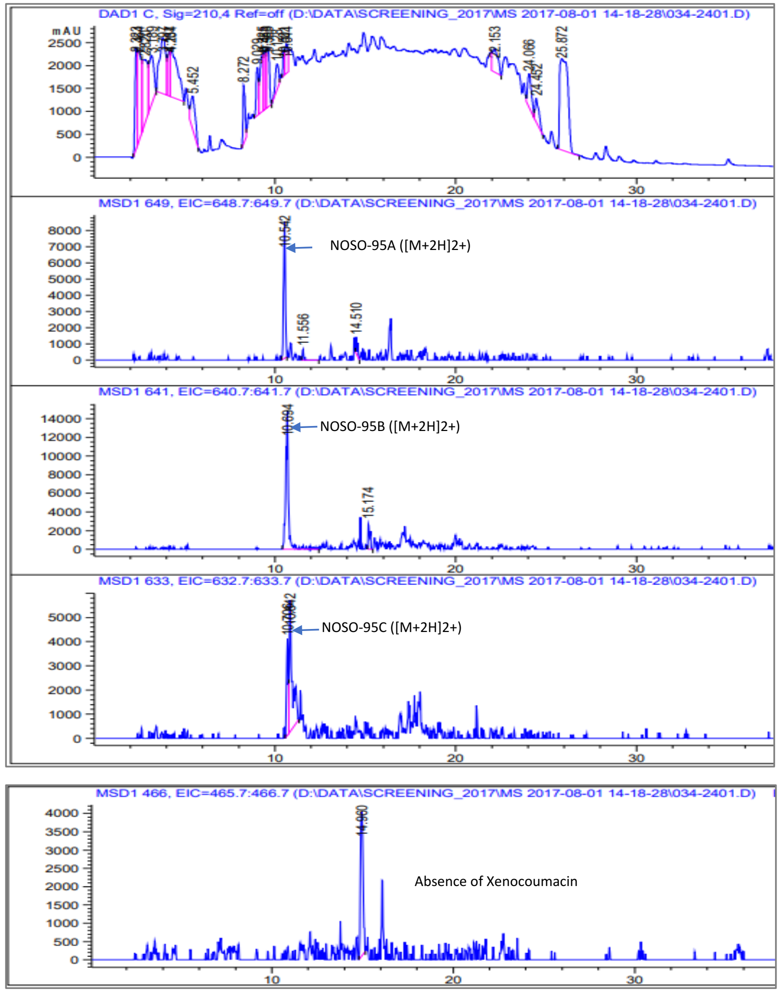

XL1 (pBB-*ngrA*) (pACYC184) + aTc

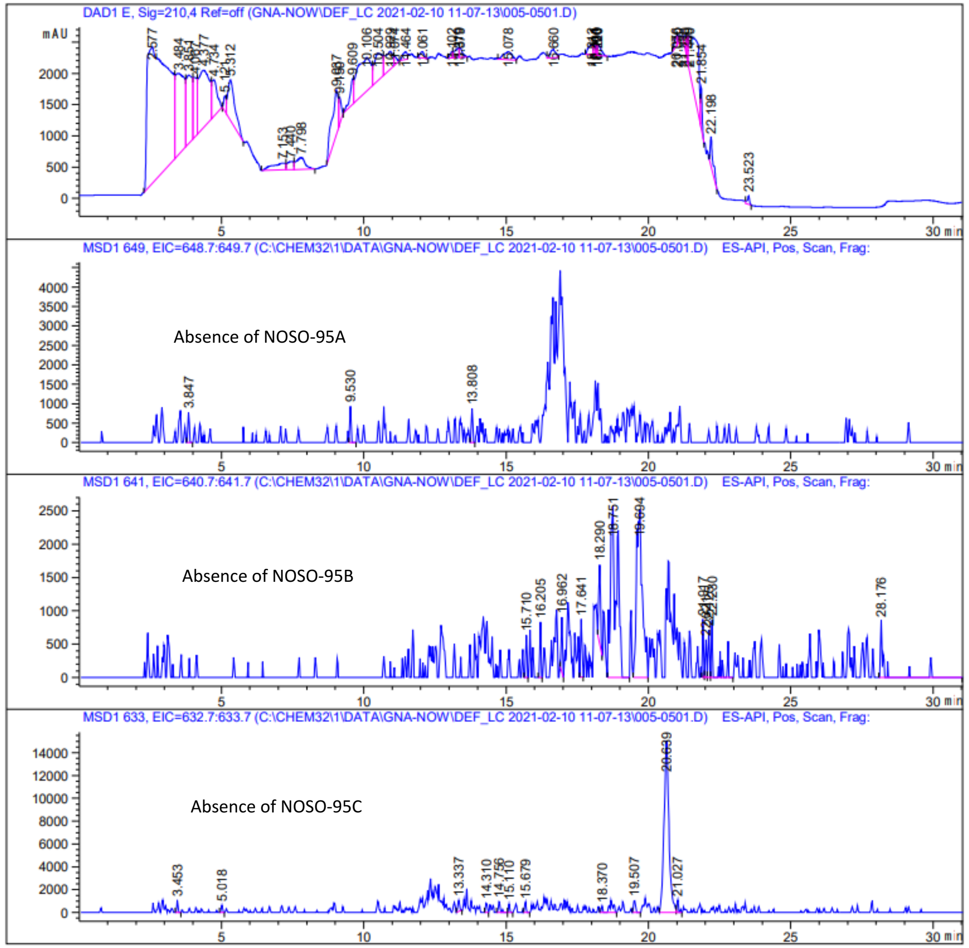

XL1 (pBB-ngrA) (p15A-P$_{tet}$-*odl*-BGC) + aTc

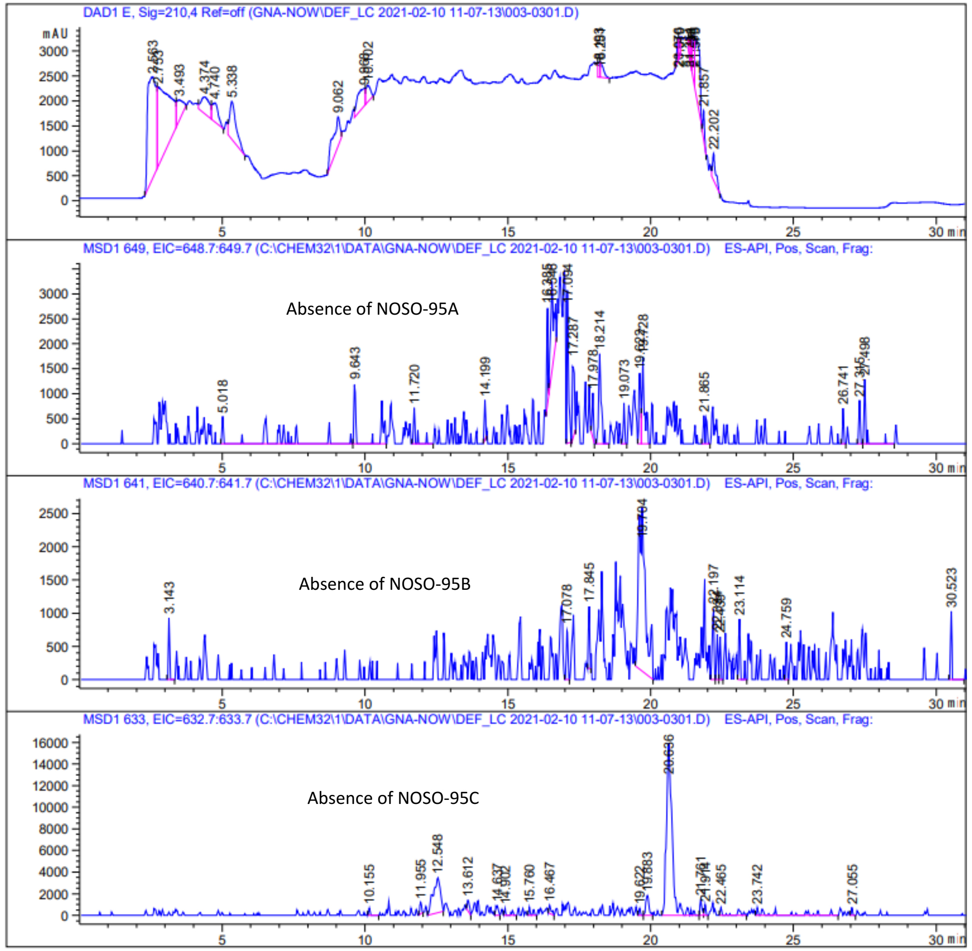

Supplement: FIG S1 [file mbio.02826-21-sf001.pdf]
