# Supplementary material for: The Odilorhabdin Antibiotic Biosynthetic Cluster and Acetyltransferase Self-Resistance Locus Are Niche and Species Specific
Source: mBio. 2022 Jan 11;13(1):e02826-21. doi: 10.1128/mbio.02826-21 (PMC8749412; doi:10.1128/mbio.02826-21)
Supplement: FIG S2 [file mbio.02826-21-sf002.pdf]

Fig. S2. Complementation of *E.coli* XL1 (pBB-*ngrA*) with p15A-P$_{tet}$-*odl*-BGC. (**A**) Expression of *odl*-BGC locus genes in XL1 (pBB-*ngrA*) (p15A-P$_{tet}$-*odl*-BGC). Total RNA was first extracted from 100 mL broth cultures of XL1 (pBB-*ngrA*) (p15A-P$_{tet}$-*odl*-BGC) or XL1 (pBB-*ngrA*) (pACYC184) incubated with or without aTc at 28°C for 78 hours, as previously described (52). Briefly, RNA was isolated with the RNeasy Protect Bacteria miniprep kit (Qiagen) including DNase I incubation in accordance with the manufacturer's recommendations. For each RNA preparation, we assessed DNA contamination by carrying out a 16S control PCR. The quantity and quality of total RNA were assessed with a NanoDrop 2000 spectrophotometer (Thermo Fisher Scientific) and an Agilent 2100 Bioanalyzer with the RNA 6000 Nano LabChip kit (Agilent). RT-qPCR was performed in two steps, as previously described (52). First, the cDNA was synthesized from 0.5 µg of total RNA with Super Script II Reverse Transcriptase from Invitrogen and random hexamers (100 mg.L^{-1}) from Promega. We then performed qPCR in triplicate with the SensiFAST SYBR® *No-ROX* kit (Bioline), with 1 µL of cDNA synthesis mixture (diluted 1:50) and 1 µM specific primers for the studied genes (Table S2). The enzyme was activated by heating for 2 minutes at 95°C. All qPCRs were performed in three technical replicates, with 45 cycles of 95°C for 5 s, 61°C for 30 s, and were monitored in the LightCycler 480 system (Roche). Melting curves were analyzed for each reaction and each curve contained a single peak. For standard curves, the amounts of PCR products generated were determined with serially diluted genomic DNA from *X. nematophila* (for *ectB* and *odl1* genes) or *E. coli* (for *gyrB* gene). Histograms represent the relative transcript level of *ectB* and *odl1* genes versus the *gyrB* gene used as housekeeping gene, as calculated with LightCycler 480 software (Roche). The data shown are the medians of experimental triplicates and error bars represent the statistical standard deviations. (**B**) Global antimicrobial activities against *Klebsiella pneumoniae* ATCC 43816 as a target of strains harboring *odl*-BGC loci (see Methods). (**C**) Supernatants from cultures used for RNA preparation (see (**A**)) were passed through a filter with 0.2 µm pores and concentrated ten-fold for LC-MS analysis. Natural odilorhabdins (NOSO-95A, B, C) (ODLs) and xenocoumacin 1 (Xcn 1) were identified in culture supernatants by LC-MS as previously described in Materials and Methods (see also fig. S1).

A

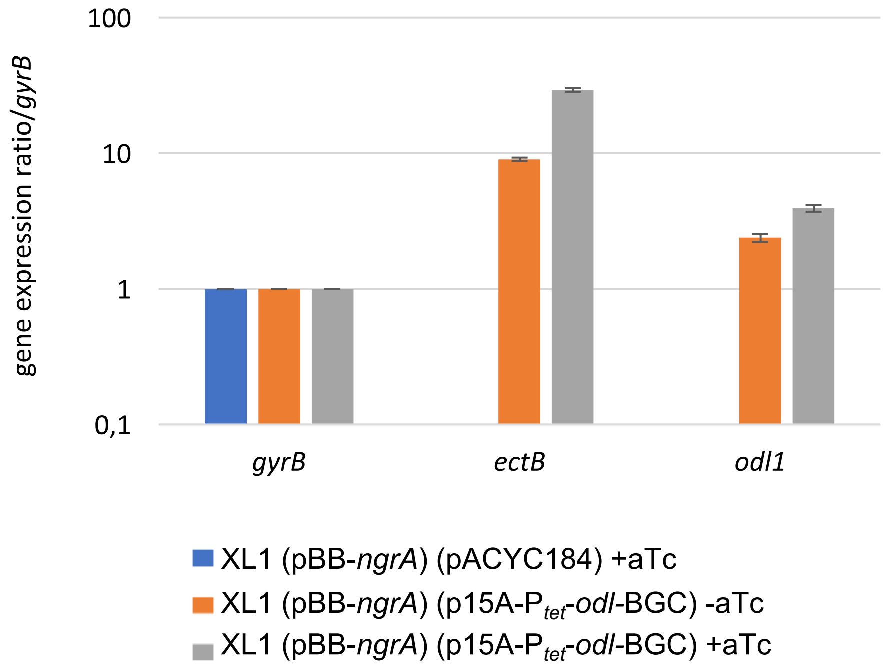

B

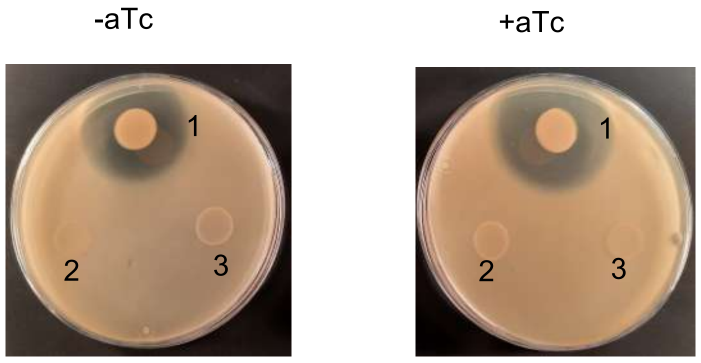

1: AN6 WT

2 : XL1 (pBB-*ngrA*) (pACYC184)

3 : XL1 (pBB-*ngrA*) (p15A-P_{tet}-*odl*-BGC)

C

Strains	Xcn 1	ODLs	***ectB* and *odl1* genes expression**
XL1 (pBB-*ngrA*) (pACYC184)*	-	-	+
XL1 (pBB-*ngrA*) (p15A-P_{tet}-*odl*-BGC) *	-	-	+

*induction with aTc
