# Supplementary material for: The Odilorhabdin Antibiotic Biosynthetic Cluster and Acetyltransferase Self-Resistance Locus Are Niche and Species Specific
Source: mBio. 2022 Jan 11;13(1):e02826-21. doi: 10.1128/mbio.02826-21 (PMC8749412; doi:10.1128/mbio.02826-21)

Fig. S3. SDS-PAGE of His-tagged OatA protein production and purification. We assessed production of the His-tagged OatA protein, by centrifuging 500 µL of a culture of the strain BL21(DE3)(pET-*oatA*) after induction with IPTG and resuspending the pellet in 20 µL of 2 x Laemmli Sample Buffer. The sample was then run on a 15% acrylamide SDS-PAGE gel (2). The BL21(DE3)(pET-28a) strain was used as a control (1). After purification of the His-tagged OatA protein with NiNTA agarose, aliquots (10 µL) of 250 mM imidazole (3) and 500 mM imidazole (4) eluates were also run on a 15% acrylamide SDS-PAGE gel with the PageRuler Plus Prestained Protein Ladder (Thermo Fisher Scientific) (M). After elecrophoresis at 150V (3W) for 5 hours, the acrylamide gel was stained overnight in a solution containing 0.1% Coomassie Blue with ethanol-acetic acid (40%-10%) and destained in ethanol-acetic acid (40%-10%) for 3 hours before the gel was scanned.

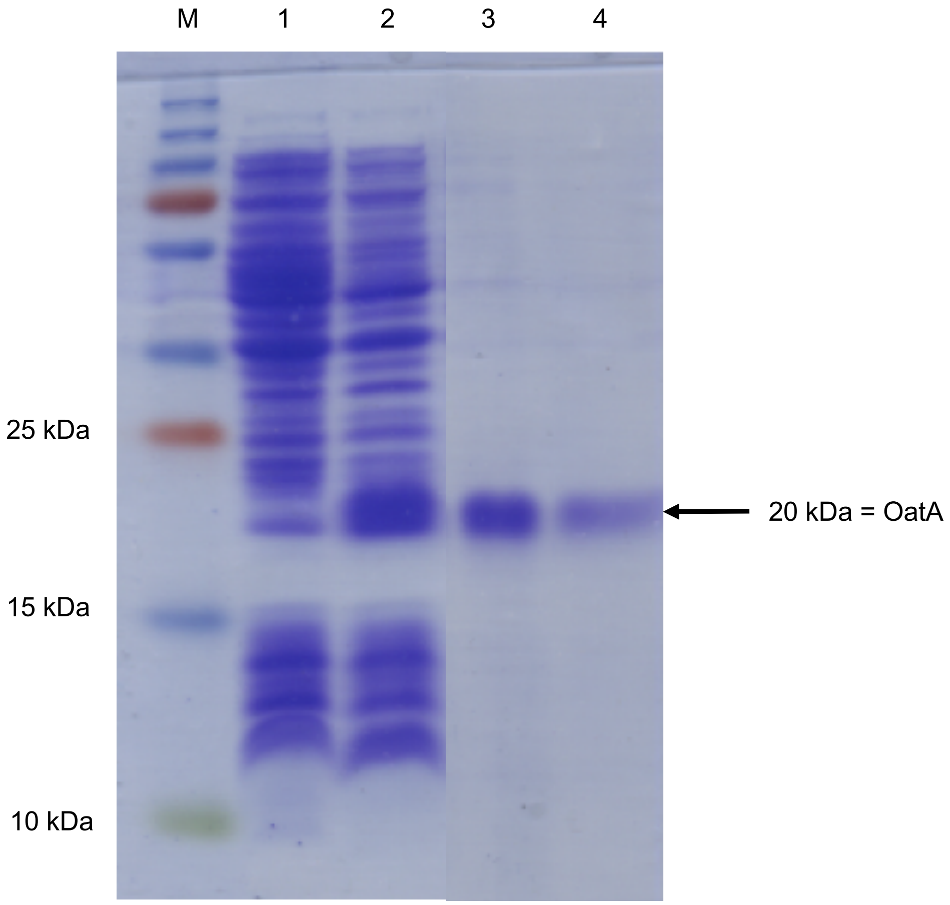

Supplement: FIG S3 [file mbio.02826-21-sf003.pdf]
