# Supplementary material for: The Odilorhabdin Antibiotic Biosynthetic Cluster and Acetyltransferase Self-Resistance Locus Are Niche and Species Specific
Source: mBio. 2022 Jan 11;13(1):e02826-21. doi: 10.1128/mbio.02826-21 (PMC8749412; doi:10.1128/mbio.02826-21)

Fig. S4. LC-MS analysis of NOSO-95C and acetylated derivatives after *in vitro* acetylation (see Materials and Methods) at different times of reaction (15 min, 4h, or 24h) with Acetyl-CoA (**B**), or Odilorhabdin acetyltransferase A (OatA) (**C**), or both (**A**). For each tested condition, a table and corresponding LC-MS chromatograms present observed ions with retention time, peak area and suggested compounds. LC-MS were performed on an Agilent 1260 Infinity HPLC system with a Waters Symmetry analytical C18 column (5 µm, 4.6 mm×150 mm). We injected 10 µL of reaction mixture into the apparatus, the flow rate was set to 0.7 mL/min and UV detection was performed at 220, 230 and 250 nm. The following mobile phases were used: A: 0.1% trifluoroacetic acid in water; B: acetonitrile. The following gradient was used: 2 to 30% of B in A from 0 to 15 min. ESI-LC-MS data were obtained in positive mode on an Agilent 1260 Infinity system (Agilent 6120 Quadrupole LC/MS, 1260 Quaternary Pump, 1260 ALS, 1260 TCC, 1260 DAD VL, 1260 FC-AS).

<u>**A:** Odilorhabdin acetyltransferase A (OatA) + acetyl-CoA + NOSO-95C (400 µg/mL)</u>

- Incubation time: 15 min

Retention time (min)	Peak Area (230 nm)	Ion mass	Proposed compound
9.71	1106	[M+H] +: 1308.7<br>[M+2H]2+: 632.8<br>[M+2H]2+: 653.9<br>[M+2H]2+: 674.8	Mono-Acetylated NOSO-95C<br>NOSO-95C<br>Mono-Acetylated NOSO-95C<br>Bi-acetylated NOSO-95C

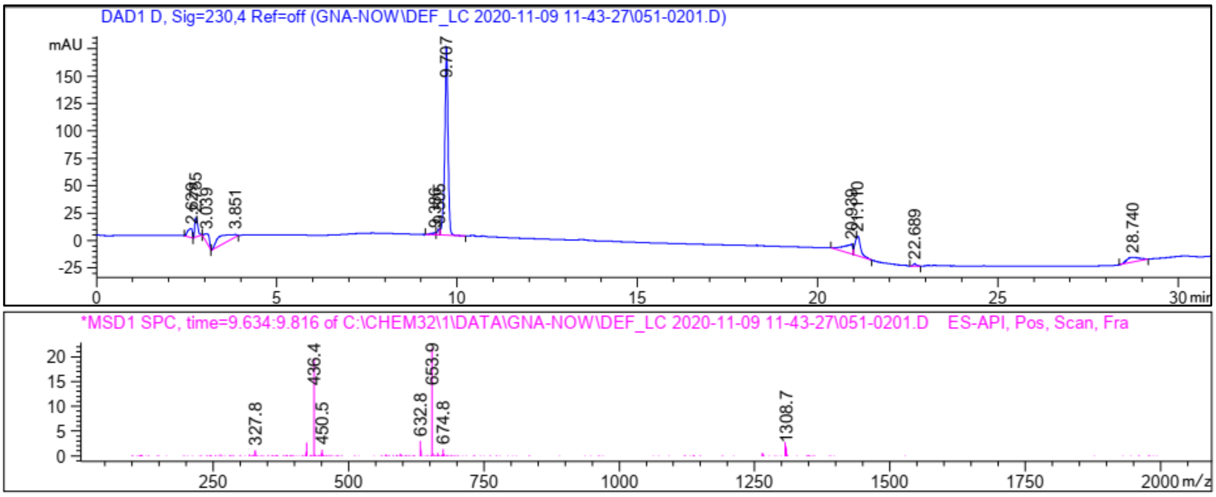

- Incubation time : 4h

Retention time (min)	Peak Area (230 nm)	Ion mass	Proposed compound
9.67	1001	[M+H]+: 1349.7<br>[M+2H]2+: 654.0<br>[M+2H]2+: 675.0	Bi-Acetylated NOSO-95C<br>Mono-Acetylated NOSO-95C<br>Bi-Acetylated NOSO-95C
10.41	40	[M+H]+: 1392.2<br>[M+2H]2+: 695.8	Tri-Acetylated NOSO-95C<br>Tri-Acetylated NOSO-95C

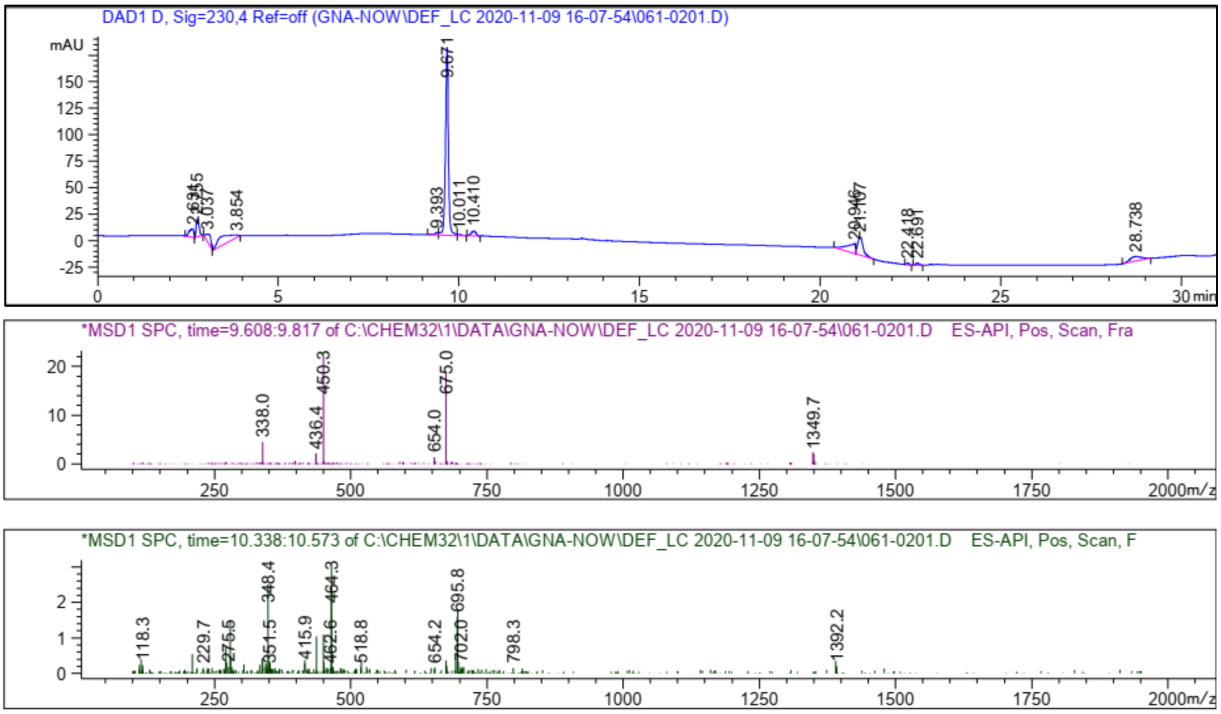

- Incubation time : 24h

Retention time (min)	Peak Area (230 nm)	Ion mass	Proposed compound
9.67	579	[M+H]+: 1349.6<br>[M+2H]2+: 674.8	Bi-Acetylated NOSO-95C<br>Bi-Acetylated NOSO-95C
10.41	137	[M+H]+: 1391.7<br>[M+2H]2+: 696.2	Tri-Acetylated NOSO-95C<br>Tri-Acetylated NOSO-95C

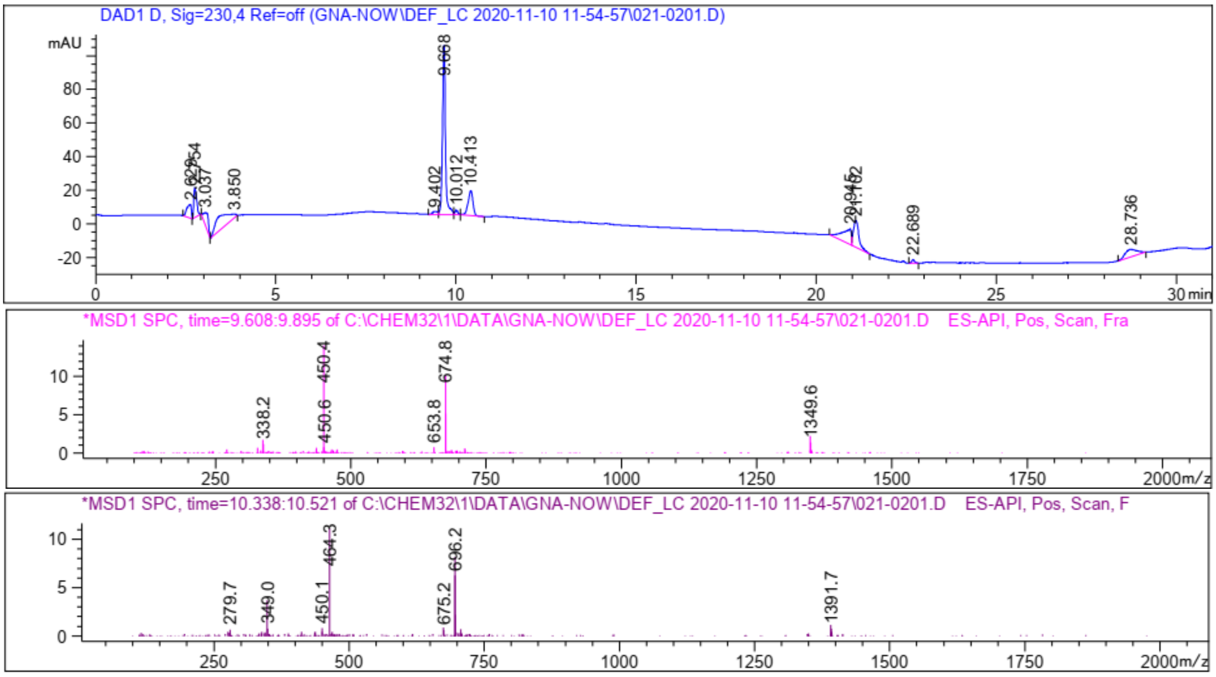

B: Acetyl-CoA + NOSO-95C (400 µg/mL)

- Incubation time: 15 min

Retention time (min)	Peak Area (230 nm)	Ion mass	Proposed compound
9.71	1139	[M+H]+: 1265.6<br>[M+2H]2+: 633.0	NOSO-95C<br>NOSO-95C

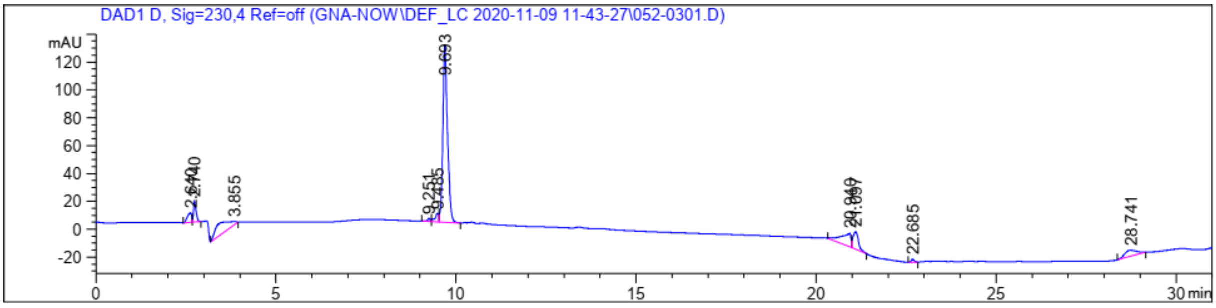

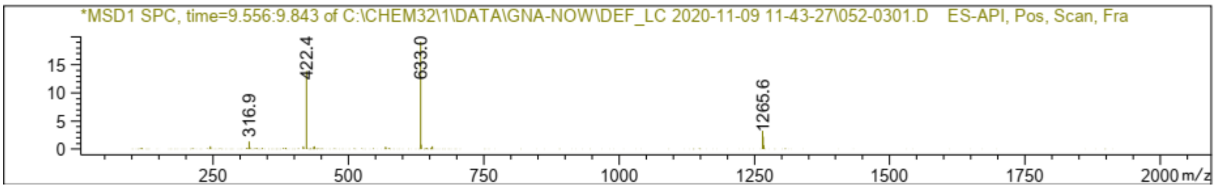

- Incubation time: 4h

Retention time (min)	Peak Area (230 nm)	Ion mass	Proposed compound
9.71	1062	[M+H]+: 1266.4<br>[M+2H]2+: 633.2<br>[M+2H]2+: 653.8	NOSO-95C<br>NOSO-95C<br>Mono-Acetylated NOSO-95C
10.36	33	[M+H]+: 1307.7<br>[M+2H]2+: 654.0	Mono-Acetylated NOSO-95C<br>Mono-Acetylated NOSO-95C

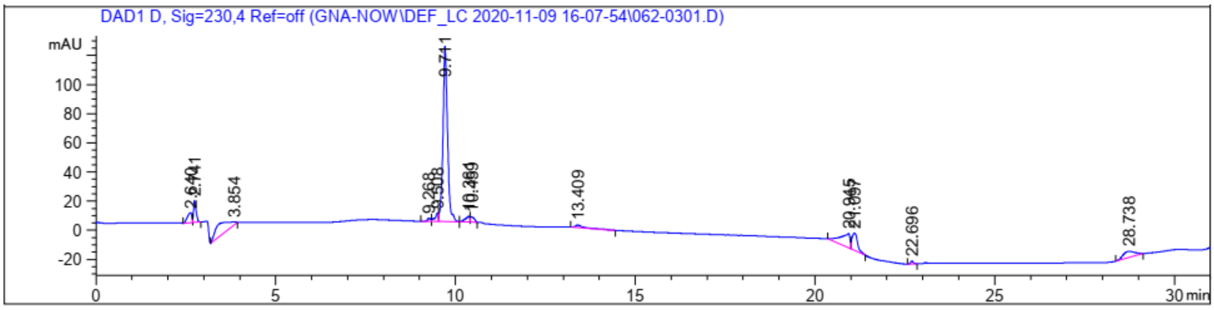

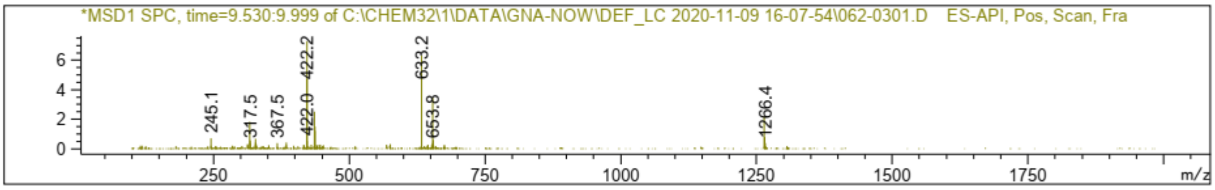

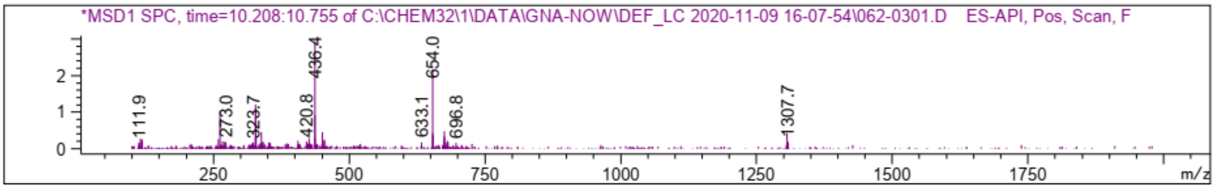

- Incubation time: 24h

Retention time (min)	Peak Area (230 nm)	Ion mass	Proposed compound
9.68	625	[M+H]+: 1266.8<br>[M+H]+: 1308.6<br>[M+2H]2+: 633.2<br>[M+2H]2+: 654.0	NOSO-95C<br>NOSO-95C<br>Mono-Acetylated NOSO-95C<br>Mono-Acetylated NOSO-95C
9.91	43	[M+H]+: 1308.6<br>[M+2H]2+: 654.3	Mono-Acetylated NOSO-95C<br>Mono-Acetylated NOSO-95C
10.33	190	[M+H]+: 1306.6<br>[M+H]+: 1348.6<br>[M+2H]2+: 655.7<br>[M+2H]2+: 675.0	Mono-Acetylated NOSO-95C<br>Bi-Acetylated NOSO-95C<br>Mono-Acetylated NOSO-95C<br>Bi-Acetylated NOSO-95C

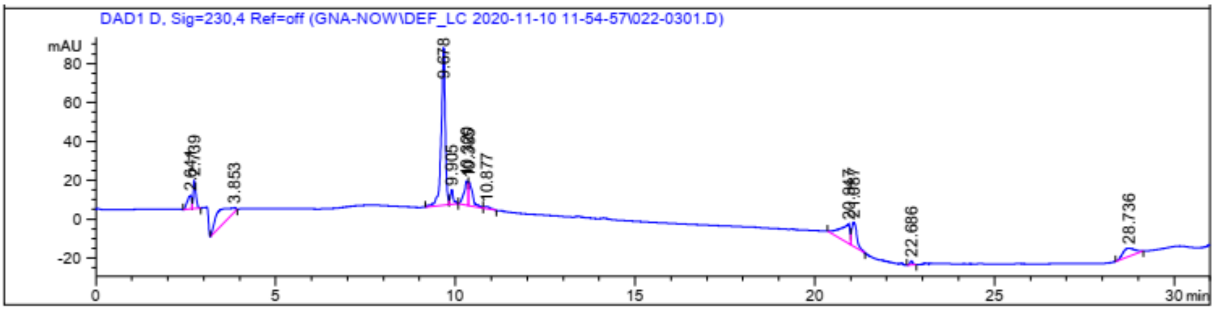

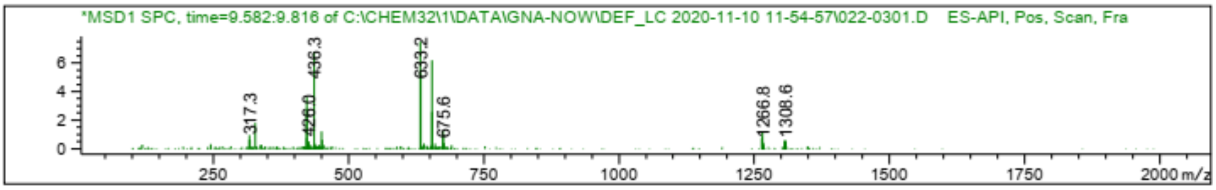

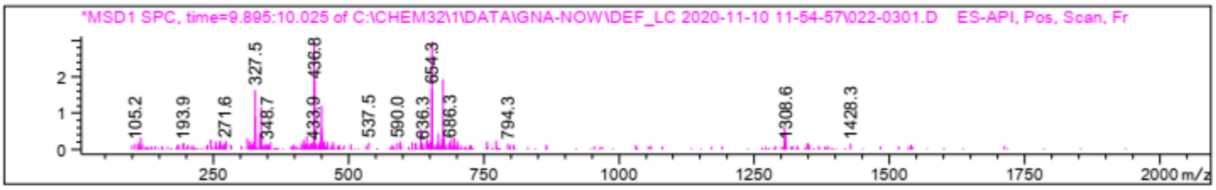

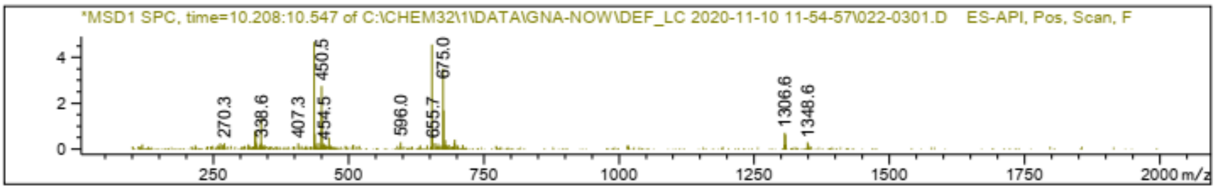

C: Odilorhabdin acetyltransferase A (OatA)+ NOSO-95C (400 µg/mL)

- Incubation time: 15 min

Retention time (min)	Peak Area (230 nm)	Ion mass	Proposed compound
9.68	1084	[M+H]+: 1265.4<br>[M+2H]2+: 633.0	NOSO-95C<br>NOSO-95C

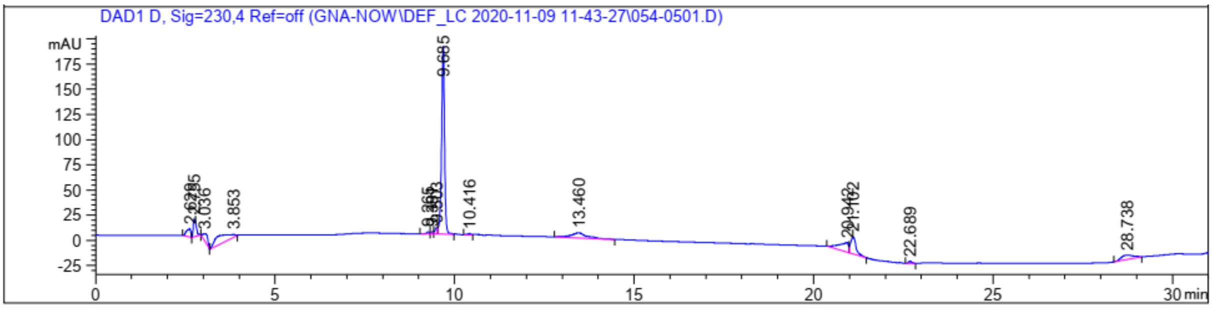

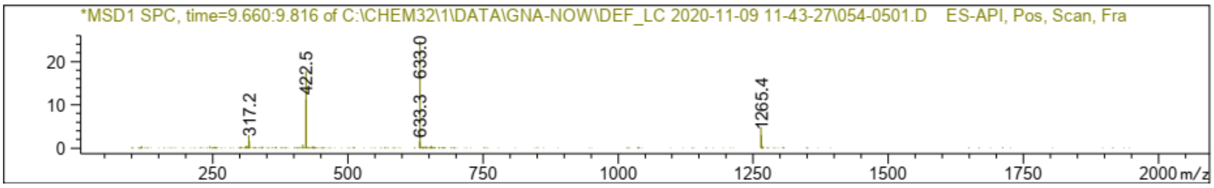

- Incubation time: 4h

Retention time (min)	Peak Area (230 nm)	Ion mass	Proposed compound
9.70	1071	[M+H]+: 1265.6<br>[M+2H]2+: 633.0	NOSO-95C<br>NOSO-95C

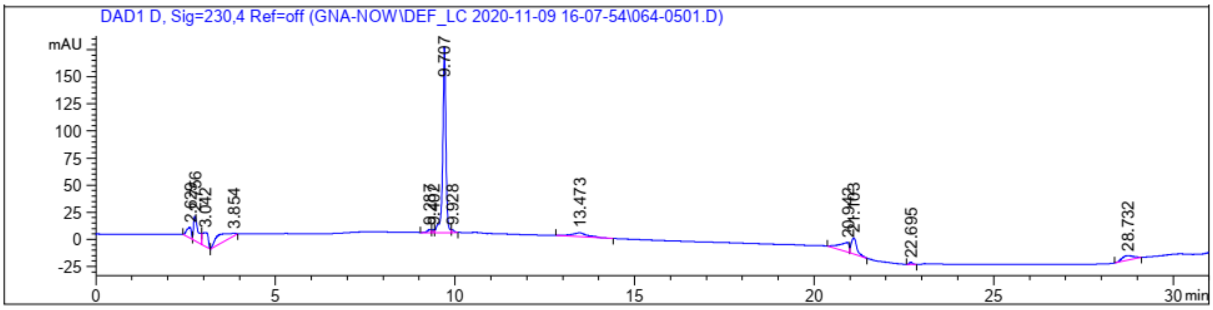

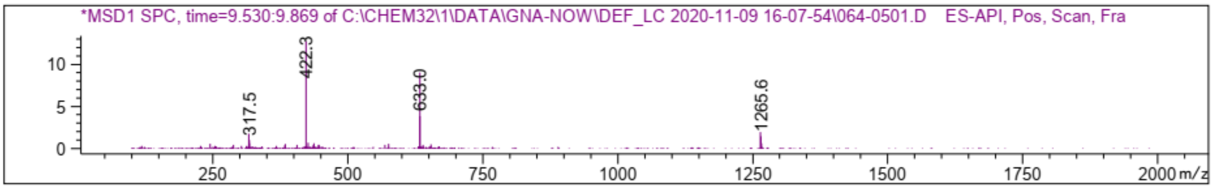

- Incubation time: 24h

Retention time (min)	Peak Area (230 nm)	Ion mass	Proposed compound
9.64	964	[M+H]+: 1265.6<br>[M+2H]2+: 633.2	NOSO-95C<br>NOSO-95C

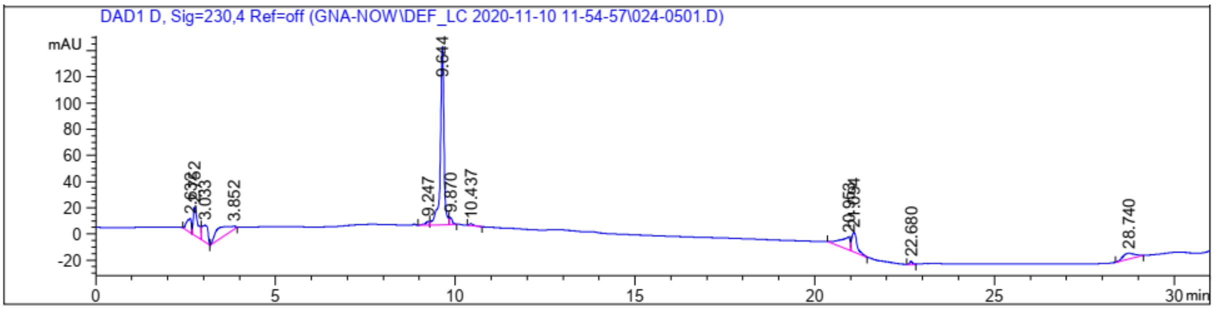

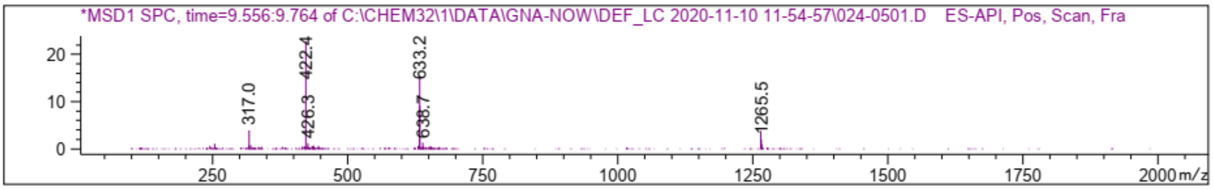

Supplement: FIG S4 [file mbio.02826-21-sf004.pdf]
