# Supplementary material for: The Odilorhabdin Antibiotic Biosynthetic Cluster and Acetyltransferase Self-Resistance Locus Are Niche and Species Specific
Source: mBio. 2022 Jan 11;13(1):e02826-21. doi: 10.1128/mbio.02826-21 (PMC8749412; doi:10.1128/mbio.02826-21)

Fig. S5. Fragmentation (MS/MS) spectrum of NOSO-95C (m/z 1265) (**A**), mono-acetylated NOSO-95C (m/z 1307) (**B**), bi-acetylated NOSO-95C (m/z 1349) (**C**), and tri-acetylated NOSO-95C (m/z 1391) (**D**). After *in vitro* acetylation, LC-MS/MS was performed as described in Methods.

A. NOSO-95C

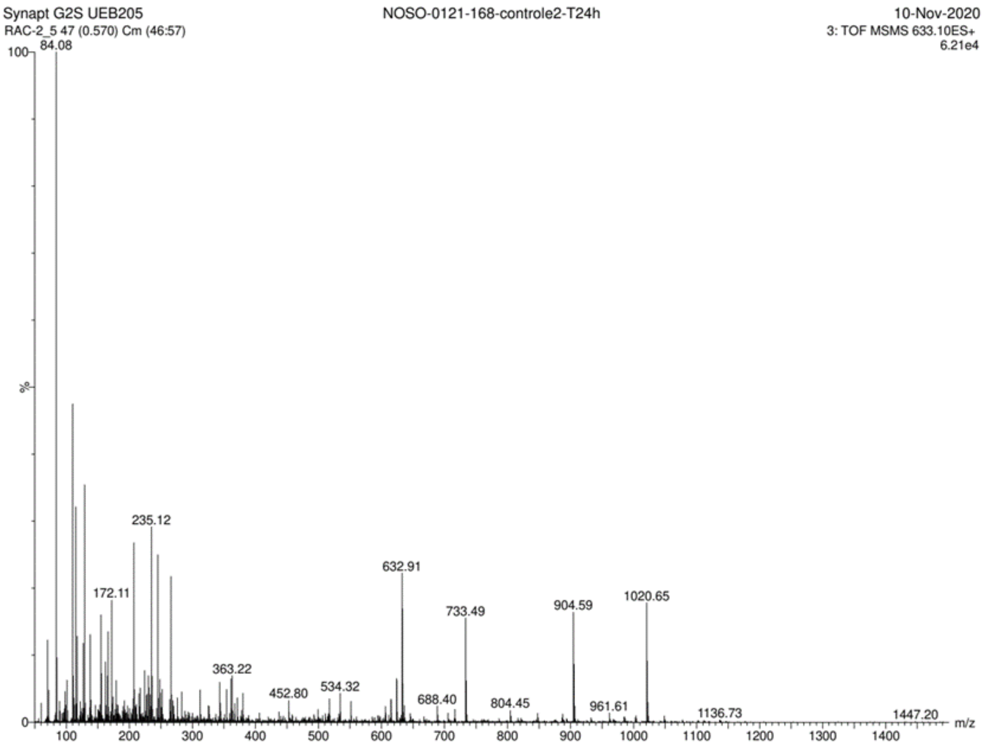

B. Mono-acetylated NOSO-95C

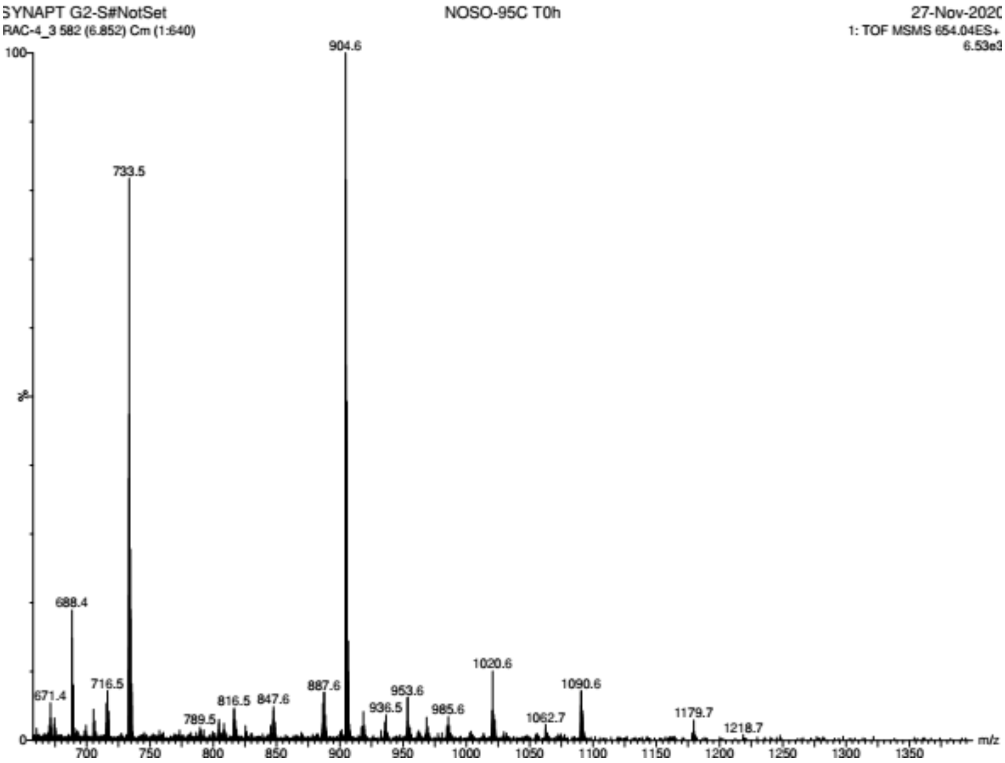

C. Bi-acetylated NOSO-95C

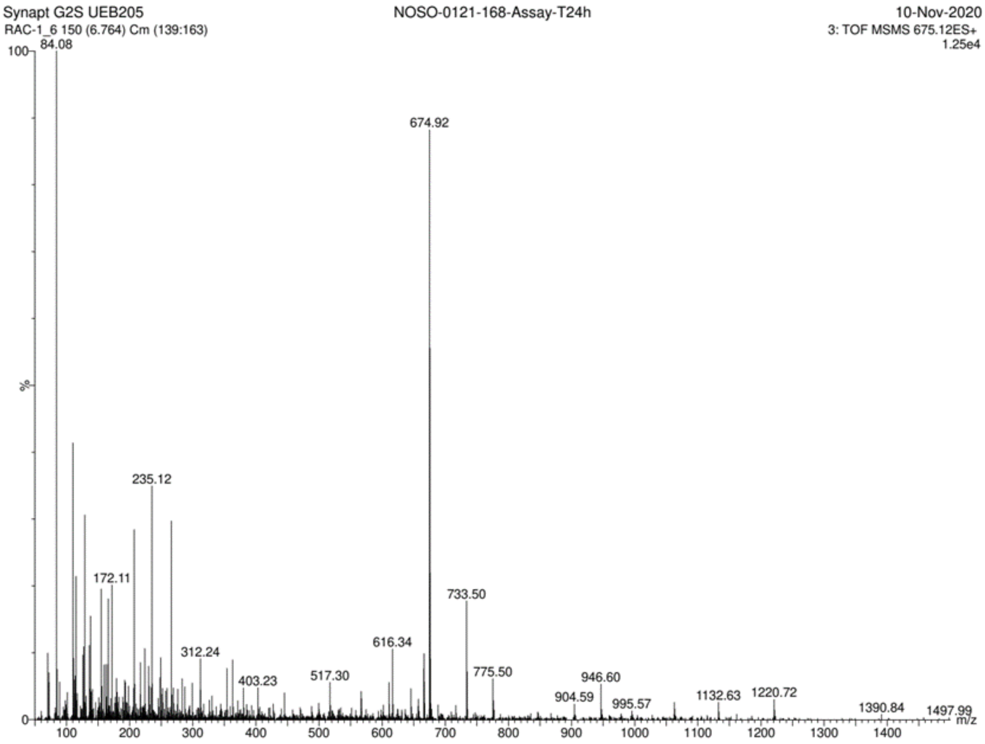

D. Tri-acetylated NOSO-95C

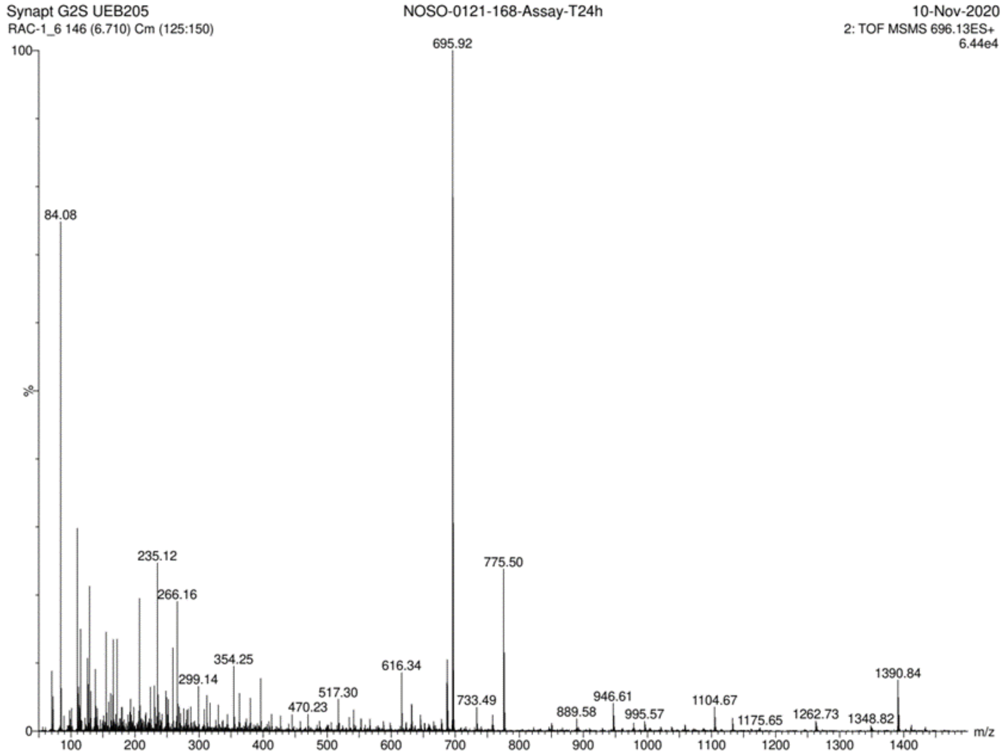

Supplement: FIG S5 [file mbio.02826-21-sf005.pdf]
