# Supplementary material for: The Odilorhabdin Antibiotic Biosynthetic Cluster and Acetyltransferase Self-Resistance Locus Are Niche and Species Specific
Source: mBio. 2022 Jan 11;13(1):e02826-21. doi: 10.1128/mbio.02826-21 (PMC8749412; doi:10.1128/mbio.02826-21)

Fig. S6. Characterization of OatA activity against a NOSO-95179 analog. (**A**) LCMS analysis of NOSO-95179 (m/z: 1022) acetylated derivatives after 24 h of reaction with acetyl-CoA and odilorhabdin acetyltransferase A (OatA): the form present was 100% mono-acetylated (m/z: 1064). (**B**) MS/MS fragmentation analysis of mono-acetylated NOSO-95179 (m/z: 1064): acetylation of the amine group on Dab(βOH)$_2$. (**C**) Antibacterial activity assay for NOSO-95179 after 24 h of reaction with acetyl-CoA (1), and OatA (2), or both (3) against *E. coli* ATCC2592. (**D**) Inhibition of synthesis of the GFP reporter protein in the *E. coli* cell-free transcription-translation system by NOSO-95179 after 24 h of reaction with acetyl-CoA , OatA, or both. Gentamicin (10 µM) was used as a positive control. The data shown are the means of three experiments ± SD.

A

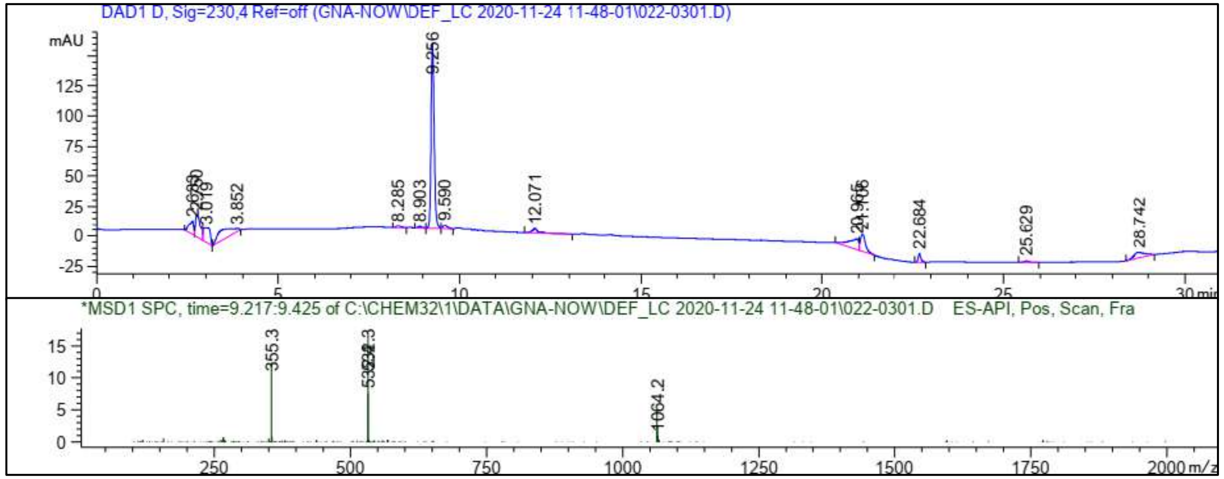

C

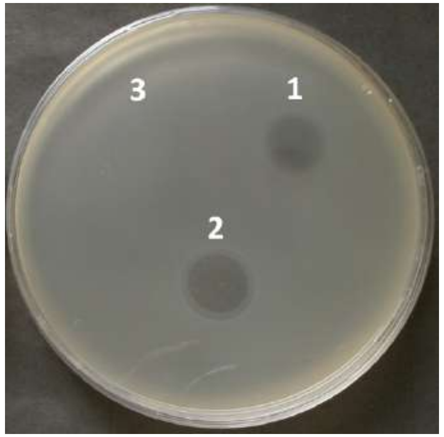

B

Ion	m/z	Neutral loss from previous ion	Attribution	Assigned Structure
[M+2H]2+	532			Lys-AcDab(βOH)-Ala-Gly-Orn-Pro-His-Lys-Dha
y_8	935.5	129	Lys_1	AcDab(βOH)-Ala-Gly-Orn-Pro-His-Lys-Dha
y_7	777.4	158	AcDab(βOH)$_2$	Ala-Gly-Orn-Pro-His-Lys-Dha
y_6	706.4	71	Ala_3	Gly-Orn-Pro-His-Lys-Dha
y_5	649.4	57	Gly_4	Orn-Pro-His-Lys-Dha
y_4	535.3	114	Orn_5	Pro-His-Lys-Dha
y_3	438.3	97	Pro_6	His-Lys-Dha

Abbreviation: Ac, acetyl group.

D

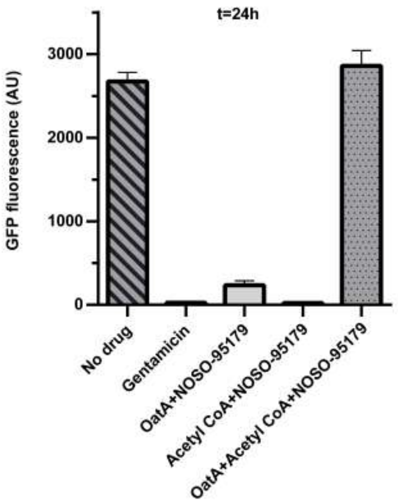

Supplement: FIG S6 [file mbio.02826-21-sf006.pdf]
