# Supplementary material for: The Odilorhabdin Antibiotic Biosynthetic Cluster and Acetyltransferase Self-Resistance Locus Are Niche and Species Specific
Source: mBio. 2022 Jan 11;13(1):e02826-21. doi: 10.1128/mbio.02826-21 (PMC8749412; doi:10.1128/mbio.02826-21)
Supplement: FIG S7 [file mbio.02826-21-sf007.pdf]

Fig. S7. Microarray analysis. Oligonucleotide probe selection and odilorhabdin microarray synthesis. Two or three 60-mer oligonucleotide probes were designed by Imaxio (Biopôle Clermont-Limagne, 63360 Saint-Beauzire, France) for each NRPS-PKS genomic module or non-module gene of the ODL-BGC from *X. nematophila* ATCC19061 and *P. laumondii* TT01. The oligonucleotide probes have a meanTm of 80.37 °C (+/-2.25°C), a mean GC% of 43.33 and no significant match elsewhere on the two bacterial genomes (less than 80% similarity over 30 nucleotides) to prevent cross-hybridization. Microarrays (8x15K) were synthesized with Agilent ink-jet technology (Agilent Technologies, Inc., Santa Clara, CA). Genomic DNA extraction, microarray hybridization and microarray image analysis. We obtained bacterial genomic DNA for 77 strains from the DGIMI laboratory collection according to the method of Brenner et al. (1982) **(1)**. For each genomic DNA, 0.5 µg of material was hydrolyzed with *Alu*I and *Rsa*I, denatured for 5 min at 95°C and Cy3-labeled with the Genomic DNA Enzymatic Labeling Kit (Agilent Technologies) according to the manufacturer's recommendations. Labeled targets were purified with Amicon 30 kDa columns (Millipore) and displayed a specific activity of 23 to 45 pmol Cy3/µg. The Cy3-labeled genomic DNA was hybridized with the 60-mer oligonucleotide microarray at 65°C for 24 hours. The arrays were scanned with Agilent Scanner (laser constant intensity) with the following settings: Dynamic AutoFocus, PMT Cy3 100%, 20 bit-data format, pixel resolution 5 µm. Microarray image files were extracted after intra-array normalization with Feature Extraction 10.7.3 software (Agilent Technologies). The mean background signal was calculated with the negative controls and subtracted from the mean signal for each spot. ODL biosynthesis gene distribution and profile clustering. After normalization and background correction, a sequence was considered present in the target genome if the hybridization signal intensity exceeded 1000 for at least one of the probes. The hybridization profiles of each strain were transformed into binary numerical values (0: gene absent; 1: gene present). The dendrogram was generated by a hierarchical analysis of the distribution patterns (presence/absence) of the ODL-targeted probes according to the unweighted pair group method arithmetic mean (UPGMA) distance method and the Gower similarity coefficient **(2)**, with Bionumerics software version 4.5 (Applied-Maths, Sint-Martens-Latem, Belgium). The taxonomic identities of the 77 strains used for hybridization experiment are shown on the right side of the dendrogram. The names of the NRPS-PKS genomic modules or non-module genes of the ODL-BGC are shown at the top of the dendrogram.

References :

1. Brenner DJ, McWhorter AC, Knutson JKL, Steigerwalt AG. 1982. *Escherichia vulneris*: a New Species of Enterobacteriaceae Associated with Human Wounds. Journal of Clinical Microbiology 15:1133–1140.
2. Gower JC, Legendre P. 1986. Metric and Euclidean properties of dissimilarity coefficients. Journal of Classification 3:5–48.

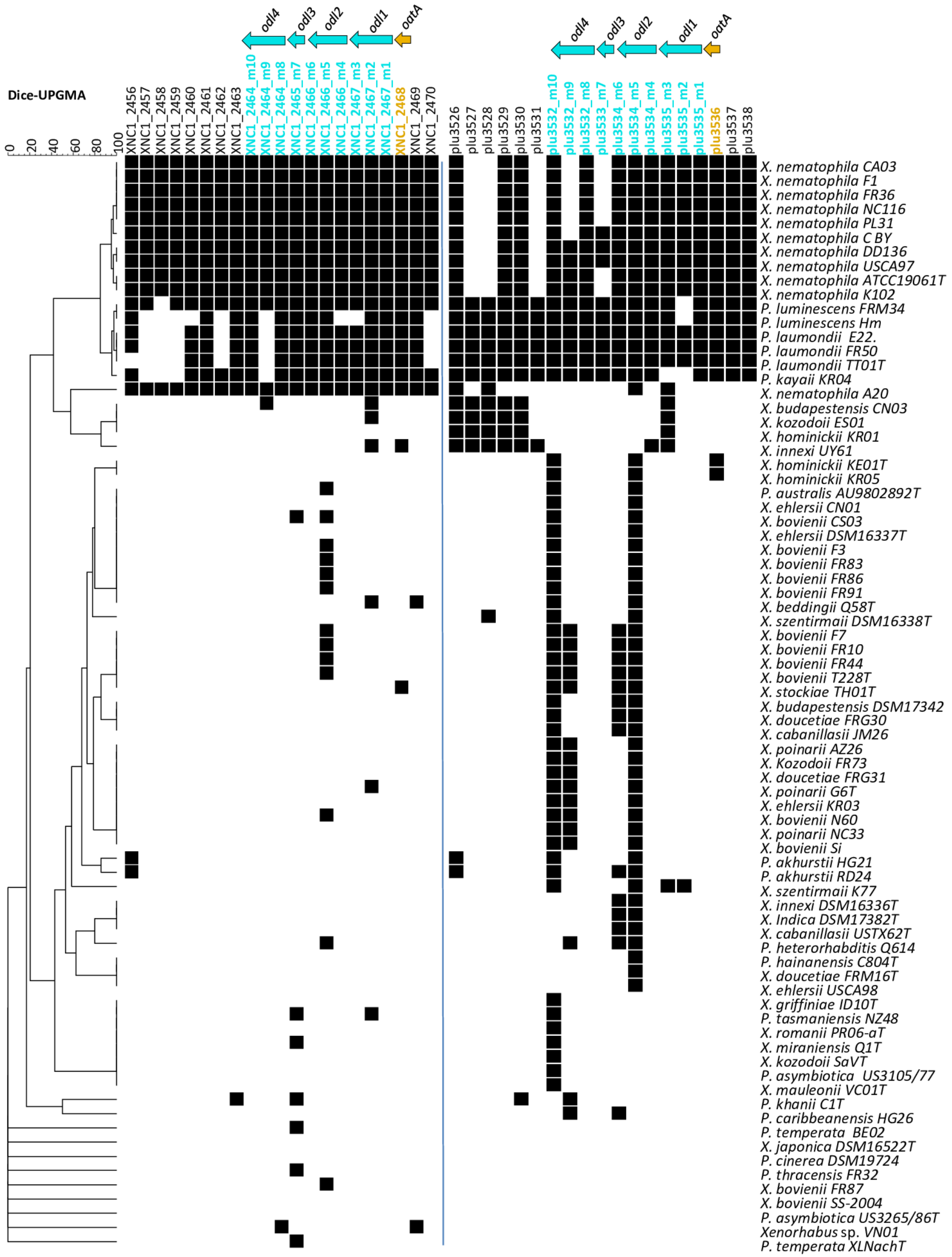

odl4
odl3
odl2
odl1
oatA
Dice-UPGMA
0
20
40
60
80
100
XNC1_2456
XNC1_2457
XNC1_2458
XNC1_2459
XNC1_2460
XNC1_2461
XNC1_2462
XNC1_2463
XNC1_2464_m10
XNC1_2464_m9
XNC1_2464_m8
XNC1_2465_m7
XNC1_2466_m6
XNC1_2466_m5
XNC1_2466_m4
XNC1_2467_m3
XNC1_2467_m2
XNC1_2467_m1
XNC1_2468
XNC1_2469
XNC1_2470
plu3526
plu3527
plu3528
plu3529
plu3530
plu3531
plu3532_m10
plu3532_m9
plu3532_m8
plu3533_m7
plu3534_m6
plu3534_m5
plu3534_m4
plu3535_m3
plu3535_m2
plu3535_m1
plu3536
plu3537
plu3538
X. nematophila CA03
X. nematophila F1
X. nematophila FR36
X. nematophila NC116
X. nematophila PL31
X. nematophila C BY
X. nematophila DD136
X. nematophila USCA97
X. nematophila ATCC19061T
X. nematophila K102
P. luminescens FRM34
P. luminescens Hm
P. laumondii E22.
P. laumondii FR50
P. laumondii TT01T
P. kayaii KR04
X. nematophila A20
X. budapestensis CN03
X. kozodoii ES01
X. hominickii KR01
X. innexi UY61
X. hominickii KE01T
X. hominickii KR05
P. australis AU9802892T
X. ehlersii CN01
X. bovienii CS03
X. ehlersii DSM16337T
X. bovienii F3
X. bovienii FR83
X. bovienii FR86
X. bovienii FR91
X. beddingii Q58T
X. szentirmaii DSM16338T
X. bovienii F7
X. bovienii FR10
X. bovienii FR44
X. bovienii T228T
X. stockiae TH01T
X. budapestensis DSM17342
X. doucetiae FRG30
X. cabanillasii JM26
X. poinarii AZ26
X. Kozodoii FR73
X. doucetiae FRG31
X. poinarii G6T
X. ehlersii KR03
X. bovienii N60
X. poinarii NC33
X. bovienii Si
P. akhurstii HG21
P. akhurstii RD24
X. szentirmaii K77
X. innexi DSM16336T
X. Indica DSM17382T
X. cabanillasii USTX62T
P. heterorhabditis Q614
P. hainanensis C804T
X. doucetiae FRM16T
X. ehlersii USCA98
X. griffiniae ID10T
P. tasmaniensis NZ48
X. romanii PR06-aT
X. miraniensis Q1T
X. kozodoii SaVT
P. asymbiotica US3105/77
X. mauleonii VC01T
P. khanii C1T
P. caribbeanensis HG26
P. temperata BE02
X. japonica DSM16522T
P. cinerea DSM19724
P. thracensis FR32
X. bovienii FR87
X. bovienii SS-2004
P. asymbiotica US3265/86T
Xenorhabus sp. VN01
P. temperata XLNachT
