# Supplementary material for: The Odilorhabdin Antibiotic Biosynthetic Cluster and Acetyltransferase Self-Resistance Locus Are Niche and Species Specific
Source: mBio. 2022 Jan 11;13(1):e02826-21. doi: 10.1128/mbio.02826-21 (PMC8749412; doi:10.1128/mbio.02826-21)

Fig. S8. Pseudogenization of the *oatA*-like gene in the genomes of the strains from *Xenorhabdus* clade C-IV.

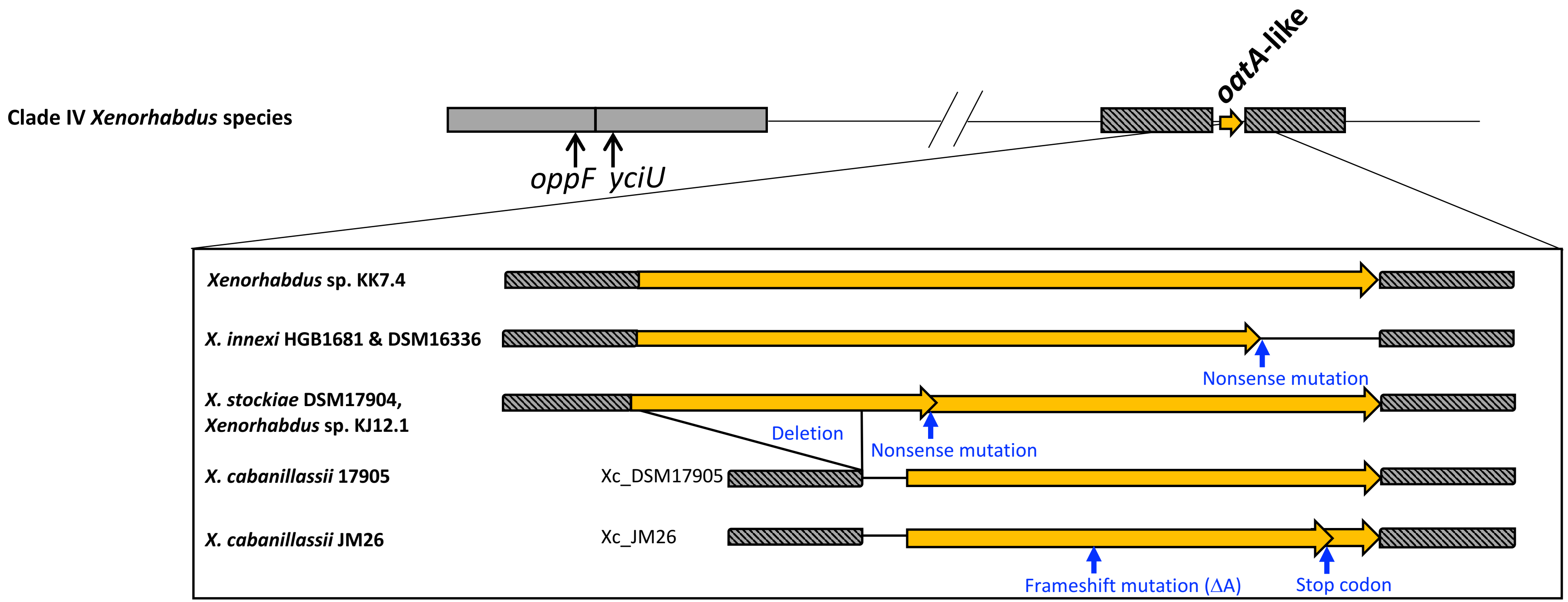

Supplement: FIG S8 [file mbio.02826-21-sf008.pdf]
