# Supplementary material for: The Odilorhabdin Antibiotic Biosynthetic Cluster and Acetyltransferase Self-Resistance Locus Are Niche and Species Specific
Source: mBio. 2022 Jan 11;13(1):e02826-21. doi: 10.1128/mbio.02826-21 (PMC8749412; doi:10.1128/mbio.02826-21)
Supplement: TABLE S1 [file mbio.02826-21-st001.pdf]

Table S1. Strains and plasmids used in this study.

Strain or plasmid	Genotype and relevant characteristics	Reference or source
**Strains**		
Xenorhabdus nematophila		
K102 (CNCMI-4530)	Wild type isolated from *Steinernema carpocapsae* nematode	Laboratory collection
AN6 WT	Wild type isolated from *Steinernema carpocapsae* nematode	(1)
Δ*xcnKL*::F	AN6/1 Δ*xcnKL* ::Km^R XNC1_2467::Cm ^{R} named also Δ*xcnKL*::*odl1*	(1)
F1	Wild type isolated from *Steinernema carpocapsae* nematode Plougastel (Brittany)	Laboratory collection
Δ*ngrA*	F1 *ngrA*::ΩCm	This study
Δ*ngrA* Δ*oatA*	F1 *ngrA*::ΩCm *oatA*::Km	This study
Escherichia coli		
GB05-dir	*E. coli* DH10B derived strain containing the arabinose inducible ETγA operon (full length *recE*, *recT*, *redγ* and *recA*) inserted at the *ybcC* locus allowing LLHR	Gene Bridges GmbH
GB08-dir	*E. coli* DH10B derived strain containing the arabinose inducible γβαA operon (full length *redγ*, *redβ*, *redα* and *recA*) inserted at the *ybcC* locus allowing LCHR	Gene Bridges GmbH
XL1-Blue MRF'	Δ(*mcrA*)183 Δ(*mcrCB-hsdSMR-mrr*)173 *endA1 supE44 thi-1 recA1 gyrA96 relA1 lac* [F′ *proAB lacIq*ZΔM15 Tn10 (Tetr)]	Stratagene
WM3064	*thrB*1004 *pro thi rpsl hsdS lac*ZΔM15 RP4-1360Δ(*araBAD*)567 Δ*dapA1341*::[erm pir (wt)] donor strain	(2)
BL21(DE3)	F$^-$ *ompT hsdSB (rB- mB-) gal dcm lon λ* (DE3 [*lacI lac*UV5-T7 gene 1 *ind1 sam7 nin5*])	Laboratory collection
**Plasmids**		
p15A-Cm	p15A origin 2kb plasmid Cm^R	(3)
p15A-*odl*-BGC	*X. nematophila* odilorhabdin locus red/ET cloned into p15A-Cm	This study
pSC101-tetR-tetO-eGFP-Km	pSC101 vector containing Km^R -*Tet*^{R} - $P_{LtetO-1}$	(3)
p15A-P_{tet}-*odl*-BGC	Km^R -Tet^R - $P_{LtetO-1}$ from pSC101-tetR-tetO-eGFP-km red/ET cloned into p15A-*odl*-BGC	This study
pACYC184	p15A origin plasmid	Laboratory collection
pBBR1-MCS5	Broad host range vector Gm^R, mob	(4)
p15A-P_{tet}-*odl*-BGC-mob	Gm^R-mob fragment from pBBR1-MCS5 red/ET cloned into p15A-P_{tet}-*odl*-BGC	This study
pJQ200SK	Gm^r *sacRB* mob oriV (p15A replicon) suicide plasmid	S. Forst
pJQ-*ngrA*::ΩCm	pJQ suicide plasmid carrying the ΩCam interposon cloned between the two regions upstream and downstream of the *ngrA* gene	This study
pBBR1-MCS2	Broad host range vector Km^R, mob	(4)
pBB-*ngrA*	*X. nematophila ngrA* gene PCR fragment cloned into pBBR1-MCS2	This study
pJQ-KmT1	pJQ200SK carrying a kanamycin resistance cassette and T1 terminator region from pPROBE-AAV	(5)
pJQ-*oatA*::KmT1	pJQ suicide plasmid carrying a kanamycin resistance cassette and T1 terminator region from pPROBE- *gfp*[AAV] cloned between the two regions upstream and downstream of the *X. nematophila oatA* gene	This study
pET-28a	pBr322 origin, T7 promoter, Kan^R	Novagen
pET-*oatA*	pET-28a with *oatA* (His-tag) in N-term under the control of T7 promoter, Kan^R	This study
pUC19	Ap^R cloning vector	Biolabs
P_{lac}-*defghij*	Seven downstream genes from *odl*-BGC-locus cloned into pUC19	This study
P_{lac}-*abc*	Three upstream genes from *odl*-BGC-locus cloned into pUC19	This study
P_{lac}-*a*	*ectB* gene (=*a* gene) cloned into pUC19	This study
P_{lac}-*b*	*b* gene cloned into pUC19	This study
P_{lac}-*oatA-Xn*	odilorhabdin acetyltransferase gene (=*c* gene) from *X. nematophila* cloned into pUC19	This study

LLHR (linear plus linear homologous recombination)
LCHR (linear plus circular homologous recombination)

References:

1. Singh S, Orr D, Divinagracia E, McGraw J, Dorff K, Forst S. 2015. Role of Secondary Metabolites in Establishment of the Mutualistic Partnership between *Xenorhabdus nematophila* and the Entomopathogenic Nematode *Steinernema carpocapsae*. Appl Environ Microbiol 81:754–764.

2. Paulick A, Koerdt A, Lassak J, Huntley S, Wilms I, Narberhaus F, Thormann KM. 2009. Two different stator systems drive a single polar flagellum in *Shewanella oneidensis* MR-1. Molecular Microbiology 71:836–850.

3. Fu J, Bian X, Hu S, Wang H, Huang F, Seibert PM, Plaza A, Xia L, Müller R, Stewart AF, Zhang Y. 2012. Full-length RecE enhances linear-linear homologous recombination and facilitates direct cloning for bioprospecting. Nature Biotechnology 30:440–446.

4. Kovach ME, Elzer PH, Steven Hill D, Robertson GT, Farris MA, Roop RM, Peterson KM. 1995. Four new derivatives of the broad-host-range cloning vector pBBR1MCS, carrying different antibiotic-resistance cassettes. Gene 166:175–176.

5. Hadchity L, Lanois A, Kiwan P, Nassar F, Givaudan A, Abi Khattar Z. 2021. AcrAB, the major RND-type efflux pump of *Photorhabdus laumondii*, confers intrinsic multidrug-resistance and contributes to virulence in insects. Environmental Microbiology Reports 13:637–648.
