# Supplementary material for: The Odilorhabdin Antibiotic Biosynthetic Cluster and Acetyltransferase Self-Resistance Locus Are Niche and Species Specific
Source: mBio. 2022 Jan 11;13(1):e02826-21. doi: 10.1128/mbio.02826-21 (PMC8749412; doi:10.1128/mbio.02826-21)
Supplement: TABLE S2 [file mbio.02826-21-st002.pdf]

Table S2. Primers used in this study

Regions or genes	Primers	Sequences 5'-3'	Length
ngrA mutant construction	L-PCR1-ngrA-XbaI	GCGCTCTAGAGGCCAGTACTTCACGATTGG	579 bp
	R-PCR1-ngrA-BamHI	GCGCGGATCCGGAACGGTCTTTGGCATTTG	
	L-ngrA2bis-BamHI	GCGGATCCCAATCGCACTGTGAACATCA	522 bp
	R-ngrA2bis-ApaI	GCGGGCCCCAGTCATTGGTGGTTTGACG	
ngrA mutant verification	L-ngrA-mut	CAGCAGCACAACAGACCAAT	6 kb
	L-PCR1-ngrA-XbaI	GCGCTCTAGAGGCCAGTACTTCACGATTGG	
ngrA gene	L-ngrA-EcoRI	GCGAATTCTCACAATCAGCAATAAAATACCAA	798 bp
	R-ngrA-BamHI	GCGGATCCTCAATAGAAGTGAATAAGAACGACTT	
oatA mutant construction	L-SacI-b	GCGAGCTCATGTCATCGCACACTTGCTC	595 bp
	R-SpeI-oatA	GCACTAGGGCATGAGCAACATATCACC	
	L-SalI-oatA	GCGTCGACGGATCACCGTCTGCTATGAG	611 bp
	R-ApaI-odl1	GCGGGCCCAGATTCCGCCTCATCTTCAC	
oatA mutant verification	R-Km-PvuI	GCCGATCG TCATTTCGAACCCCAGAGTC	1791- bp
	L-*b*-KpnI	GGCGGTACCCCGAAAGTCAAACCCAGAGA	
	M13-F	GTAAAACGACGGCCAG	3 kb
	R-*odl1*-F1q	TTCTACAGAAATATCGAAGCTGACC	
p15A-*odl*-BGC Red/ET cloning (LLHR)	odl-bis1	TTGTTCTGGTTTGGAACGATGATACTAACATCAACCCCCCGCATTGCGGCTGTACAAATGGTTACGCCCCGCCCTGCCACTC	44.5 kb
	odl-bis2	CGGAAATGTTCGACAATATGAACGAGCTTTTGACTCATAATTATTGAATATCTCTTCCACGGTAACGAATCAGACAATTGACG	
p15A-P_{tet}-*odl*-BGC Red/ET cloning (LCHR1)	p15A-odilo-5	TCTGCAAACCCTATGCTACTCCGTCAAGCCGTCAATTGTCTGATTCGTTACCTAATGCGGTAGTTTATCACA	2093 bp
	p15A-odilo-3	AAATGTTCGACAATATGAACGAGCTTTTGACTCATAATTATTGAATATCTCTTCCACATGAATTCCTCCTGCTAGAT	
p15A-P_{tet}-*odl*-BGC-mob Red/ET cloning (LCHR2)	L-Gm-Km	ATAAATAAAATGGCGCCACAGGTGCGGTTGCTGGCGCCTCGACCCAAGTACCGCCAGAGAGGCGGTTTGCGTATT	2786 bp
	R-oriV-Ptet	CCGTTCGTATAGCATACATTATACGAAGTTATTCGGCTTGAACGAATTGTTAGGGGGGCTTGTTGATCGACTG	
defghij region	L-d-KpnI	GGCGGTACCTTTATGGGCGGTTGGTGTAT	7205 bp
	R-j-SacI	GGCGAGCTCATTGGTTTAGCGGCATCGGA	
abc region	L-*ectB*-KpnI	GGCGGTACCAAAAAGAGGTTAAAGTGGAAGAGA	2764 bp
	R-c-SacI	GGCGAGCTCTTCAAGTTACGGCTTCGTTG	
ectB gene	L-*ectB*-KpnI	GGCGGTACCAAAAAGAGGTTAAAGTGGAAGAGA	1395 bp
	R-*ectB*-SacI	GGCGAGCTCTGCGATGACATTCATTCTGA	
b gene	L-b-KpnI	GGCGGTACCCCGAAAGTCAAACCCAGAGA	999 bp
	R-b-SacI	GGCGAGCTCGACACATCACATCTTCATAGTTGTC	
oatA gene	L-c-KpnI	GGCGGTACCCCGTCCGTACCTTAATTATATTTCT	591 bp
	R-c-SacI	GGCGAGCTCTTCAAGTTACGGCTTCGTTG	
pET-*oatA*	F-Nterm-*oatA*-NdeI	GGCCATATGAAAGTAACTTTGACTGATGTG	478 bp
	R-Stop-*oatA*-SalI	GGCGTCGACTTGGGTATAGGATCATTAAA	
oatA Photorhabdus	L-oatA-photo-KpnI	GGCGGTACCGTATCTTAATTATATTTCTTAAA	592 bp
	R-oatA-photo-SacI	GGCGAGCTCGGAATAACAAGCGGTTCAACTT	
oatA-like X. hominickii	L-oatA-xh-KpnI	GGCGGTACCTCAAAGTATCTTCCGCACCTT	589 bp
	R- oatA-xh-SacI	GGCGAGCTCATAAGCGGGAGAGGCAGTTT	
oatA-like X. innexi	L-oatA-xi-KpnI	GGCGGTACCTCAATAAACTGATGGCAATTACA	473 bp
	R- oatA-xi-SacI	GGCGAGCTCCATTTCTTCATGCTCATCACAA	
oatA-like X. cabanillassii	L-oatA-xc-KpnI	GGCGGTACCTGTGATTCAAAGGGAGAGCA	430 bp
	R- oatA-xc-SacI	GGCGAGCTCATTCTTTTCGCAAGCTCCAG	
oatA-like X. stockiae	L-oatA-xs-KpnI	GGCGGTACCTGGCTATTACATTGTGATTATGGA	527 bp
	R- oatA-xs-SacI	GGCGAGCTCTGTTCATTTTTAAAGAGAAGTTCACA	

In red : Forward homology arm
In blue : Reverse homology arm

Regions or genes	Primers	Sequences 5'-3'	used for
gyrB	F-*gyrB*-Ecq	ACCCGAATATCTTCTACTTCTCCAC	qPCR
	R- *gyrB*-Ecq	CTGTAGCCTTCTTTGTCCATGTAG	
ectB	F-*ectB*-F1q	TCTGGGCTATATTCTCAAATCACAC	qPCR
	R-*ectB*-F1q	CGCCTTGTACAGTCTCAAGAATAAC	
odl1	F- *odl1*-F1q	ATCAGAACATCTGGCTTATGTGATT	qPCR
	R- *odl1*-F1q	TTCTACAGAAATATCGAAGCTGACC	
